# Global, Regional, and National Burdens of Neck Pain in Adolescents and Young Adults Aged 10–24 Years From 1990 to 2021: A Population‐Based Study

**DOI:** 10.1155/prm/3439180

**Published:** 2026-07-23

**Authors:** Shu Qian, Sheng-Jie Guo, Yang Song, Hao-Ran Gao, Hua Hui, Xiong Niu, Fei-Long Wei, Yi-Fang Yuan

**Affiliations:** ^1^ Department of Spine Surgery, Honghui Hospital, Xi’an Jiaotong University, Xi’an 710054, Shaanxi, China, xjtu.edu.cn; ^2^ Department of Orthopaedics, Tangdu Hospital, Fourth Military Medical University, Xi’an 710038, China, fmmu.edu.cn; ^3^ Department of Orthopaedics, The First Affiliated Hospital of Xi’an Medical University, Xi’an 710077, China, xjtu.edu.cn; ^4^ Department of Orthopaedics, General Hospital of Central Theater Command (Wuhan General Hospital of Guangzhou Command, Previously), 430030, Wuhan, China

**Keywords:** adolescent, neck pain, young adults

## Abstract

**Background:**

Neck pain is a prevalent condition that poses substantial health challenges for adolescents and young adults worldwide. However, data on its burden and trends in this population are limited. This study aimed to evaluate trends in the burden of neck pain among adolescents and young adults aged 10–24 years from 1990 to 2021 at the global, regional, and national levels.

**Materials and Methods:**

This trend analysis, derived from the Global Burden of Diseases, Injuries, and Risk Factors Study 2021, aimed to estimate the incidence, prevalence, and years lived with disability (YLDs) related to neck pain in individuals aged 10–24. We reported case counts, rates per 100,000 individuals, and average annual percentage changes (AAPCs). Furthermore, global trends were further analyzed by age, sex, and the sociodemographic index (SDI).

**Results:**

Globally, the prevalence of neck pain among adolescents and young adults decreased from 1029 (95% uncertainty interval [UI]: 556–1755) in 1990 to 1019 (95% UI: 549–1742) per 100,000 in 2021, with an AAPC of −0.05 (95% confidence interval [CI] from −0.07 to −0.03, *p* = 0.001). Those aged 10–14 years experienced a significant increase in prevalence (from 496 [95% UI: 272–1381] to 500 [95% UI: 275–1365] per 100,000, with an AAPC of 0.03 [95% CI: 0.01–0.04], *p* = 0.001). The YLDs decreased from 107 (95% UI: 52–200) in 1990 to 106 (95% UI: 51–197) per 100,000 in 2021, with an AAPC of −0.05 (95% CI: −0.08 to −0.02, *p* = 0.001). Globally, the prevalence is higher in females (1182 [95% UI: 638–1365]) than in males (863 [95% UI: 459–1365]), with the peak among those aged 20–24 years. Tropical Latin America had the highest prevalence and YLDs from 1990 to 2021. By the SDI quintile, high‐income countries had the highest prevalence and YLDs of neck pain during the study period.

**Conclusion:**

Globally, the age‐standardized prevalence rate of neck pain among adolescents and young adults declined from 1990 to 2021, and this trend was unaffected by the COVID‐19 pandemic. Although the prevalence among young adults shows a downward trend, neck pain remains a considerable burden. Notably, the prevalence of neck pain among adolescents aged 10–14 years continued to increase. This study provides insights for evidence‐based resource allocation for neck pain screening, prevention, and intervention strategies targeting adolescents and young adults.

## 1. Introduction

Neck pain, whether caused by traumatic or nontraumatic, is a common and frequently debilitating condition that results in significant self‐reported pain, disability, and a sustained burden on personal well‐being and healthcare resources [1, 2]. It is particularly prevalent among individuals in desk‐bound occupations and ranks among the leading causes of disability worldwide [3, 4]. The causes of neck pain are multifactorial, encompassing low levels of physical activity [5, 6], inflammatory and degenerative processes of the cervical spine [7, 8], and noncatastrophic injuries resulting from sports‐related activities [5] or other degenerative diseases [7]. However, the Global Burden of Diseases, Injuries, and Risk Factors Study (GBD) 2021 has not clearly delineated the causes and related risk factors of neck pain within its population estimates.

Accurate prevalence estimates are essential for etiological studies, healthcare evaluations, and assessments of the impact of neck pain on the general population [9]. Adolescents and young adults aged 10 to 24—a considerable segment of the study‐age and working population—are at an increased risk of experiencing neck pain. This can negatively affect their productivity and require ongoing healthcare services during the early stages of the disease, placing a burden on caregivers and adding financial challenges to their daily lives. Each version of the GBD highlights the overall impact of musculoskeletal disorders, focusing specifically on neck pain [2–4].

A recent study by Fu et al. examined the prevalence trends of neck pain among individuals aged 10–24 years from 1990 to 2019 using GBD data, reporting an overall decreasing trend with notable regional and sex differences [10]. While that study provided valuable insights, several important gaps remain. First, its data period ended in 2019, leaving contemporary trends—including the potential impact of the COVID‐19 pandemic—unexamined. Second, Fu et al. focused exclusively on prevalence, whereas a comprehensive understanding of disease burden requires analysis of incidence and years lived with disability (YLDs) as well. Third, their analytical approach relied on average annual percentage changes (AAPCs), without employing joinpoint regression to identify significant inflection points in temporal trends or decomposition analysis to quantify the contributions of population growth, aging, and epidemiological changes to the burden.

To address these gaps, our study provides updated estimates for 1990–2021 (including the COVID‐19 pandemic period) for incidence, prevalence, and YLDs due to neck pain among individuals aged 10–24 years at global, regional, and national levels. We apply joinpoint regression to identify years with the most significant trend changes and decomposition analysis to better understand the drivers of YLDs. By doing so, we aim to offer a more complete and current evidence base to inform targeted prevention and intervention strategies for this vulnerable population.

## 2. Methods

### 2.1. Study Population and Data Source

The data in this study were extracted from the Global Health Data Exchange Query Tool (https://ghdx.healthdata.org/gbd-results). Therefore, ethical approval was not required for the study. This study examined a population of adolescents and young adults aged 10–24, using data from the 2021 GBD public database [4]. According to the GBD case definition, neck pain is defined as discomfort localized to the cervical spine region lasting for at least 1 day, regardless of whether it radiates to one or more upper limbs [2]. The neck encompasses the region extending from the occipital bone down to the first thoracic vertebra. Data on neck pain were obtained from three distinct age ranges (10–14, 15–19, and 20–24 years) across 21 geographically close and epidemiologically similar regions grouped by countries as outlined in the GBD [4]. Adolescence is a transition between childhood and adulthood, marked by biological development and social and behavioral transformations. The World Health Organization (WHO) defines adolescents as aged between 10 and 19 years [11]. In this study, we adopted the same age range for adolescents, with young adults defined as those between 20 and 24 years. The GBD 2021 is also calculated for each country’s SDI, a composite indicator reflecting the social and economic conditions that affect health outcomes in various regions. The SDI is categorized into five quintiles: low, low‐middle, middle, high‐middle, and high.

The incidence, prevalence, YLDs cases, and associated rates are derived from GBD 2021. Rates are presented per 100,000 individuals. The 95% uncertainty interval (UI) is determined by the 25^th^ and 975^th^ percentiles from the 1000 estimates derived from the GBD algorithm. The methodology employed in GBD 2021 has been detailed in a previous study [4]. The review board at Honghui Hospital concluded that approval was unnecessary for this study since it used publicly accessible data. Furthermore, this study adhered to the Guidelines for Accurate and Transparent Health Estimates Reporting Guidelines designed for accurate and transparent reporting of health estimates in cross‐sectional studies.

### 2.2. Statistical Analysis

Initially, this study first investigates global trends in neck pain incidence, prevalence, and YLDs. Age‐specific rates and their AAPCs were calculated through linear regression, utilizing logarithmic scales for rates as the dependent variable and the years as the independent variable [12]. The AAPC is a composite indicator reflecting trends over a predetermined time frame [13]. In joinpoint regression models, the AAPC is also calculated as a weighted average of annual percentage changes (APCs), with weights corresponding to the duration of the APC interval [12]. The AAPC can be quantified numerically to reflect the mean APC across the designated years, and we determined the AAPC for these intervals: 1990–1999, 2000–2009, 2010–2021, and 1990–2021. Unlike traditional linear regression that assumes a constant trend over the entire time series (e.g., estimating a single APC for the whole period), joinpoint regression allows trend changes at specific inflection points, thereby capturing nonmonotonic patterns such as periods of increase followed by decrease. This approach provides a more nuanced understanding of temporal dynamics and avoids masking transient but significant trend reversals.

The second goal was to determine the years in which the trends showed the most notable variations. Joinpoint regression analysis was utilized to identify trends in the data over time, employing the most straightforward model by linking various line segments on a logarithmic scale. Additionally, the Monte Carlo permutation test was applied to determine the optimal joinpoint regression model, particularly the number of joinpoints. We set the maximum number of potential joinpoints to 5 (*k*_max_5) and the minimum to 0 (*k*_min_ = 0). The permutation test starts from *k*_min_ = 0; if *k* ≠ *k*_max_, it sets *k* = *k* + 1 and continues the test until the corresponding model with *k* = *k*_max_ is selected as the optimal model.

The third aim was to categorize global trends based on age group, gender, and SDI and to detail these trends at both regional and national levels. The methodology employed for AAPCs was consistent with the approach described above. To better explain the impact of underlying factors on the epidemiology of YLDs, a decomposition analysis was conducted [14, 15]. We report and interpret statistical test results with effect sizes and confidence interval (CI), rates, UI, and *p* values. RStudio software (version 4.2.1) was used for statistical analyses. And the work was reported in line with the STROCSS criteria [16].

## 3. Results

### 3.1. Global Trends

**FIGURE 1:**
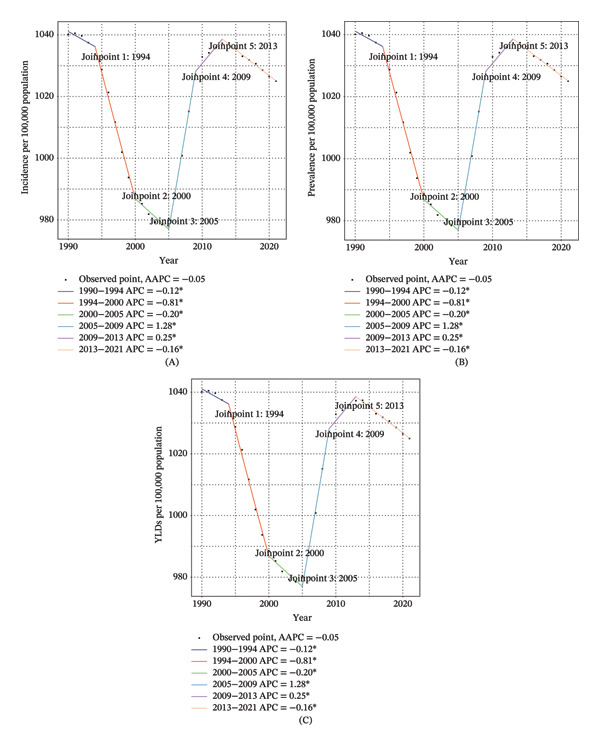
Joinpoint regression analysis of global neck pain. (A) Joinpoint regression analysis of global neck pain incidence in adolescents and young adults aged 10–24 years from 1990 to 2021. (B) Joinpoint regression analysis of global neck pain prevalence in adolescents and young adults aged 10–24 years from 1990 to 2021. (C) Joinpoint regression analysis of global neck pain YLDs in adolescents and young adults aged 10–24 years from 1990 to 2021. APC = annual percentage change and YLDs = years lived with disability.

Globally, the incidence of neck pain among adolescents and young adults decreased from 1990 to 1999 (AAPC –0.52 [95% CI: −0.56 to −0.48], *p* = 0.001), followed by an increase from 2000 to 2009 (AAPC 0.35 [95% CI: 0.29–0.41], *p* = 0.001) and continued to decline from 2010 to 2021, albeit at a slower rate (AAPC –0.02 [–0.05 to 0.02], *p* = 0.337), as presented in Table 1 and Figure 1A. Overall, the age‐standardized incidence rate of neck pain in 2021 (311 [95% UI: 137–561]) was lower than the incidence rate in 1990 (317 [95% UI: 138–575]) per 100,000, indicating an AAPC of −0.08 [ 95% CI: −0.11 to −0.05, *p* = 0.001] (Supporting Table 1). The incidence of neck pain in 204 countries and regions is indicated in Figure 2A. The United States, Russian Federation, Brazil, etc. have higher incidence rates (Figure 2A). In addition, the AAPC of incidence across 204 countries and regions is depicted in Figure 3A. Brazil also showed a high AAPC for incidence (Figure 3A).

**TABLE 1:** Global AAPCs in prevalence, incidence, and YLDs of neck pain in adolescents and young adults aged 10–24 years in 2021.

	Incidence		Prevalence		YLDs	
	AAPC (95% CI)	*p* value	AAPC (95% CI)	*p* value	AAPC (95% CI)	*p* value
*Period*						
1990–1999	−0.52 (−0.56 to −0.48)	0.001	−0.5 (−0.54 to −0.47)	0.001	−0.5 (−0.54 to −0.46)	0.001
2000–2009	0.35 (0.29–0.41)	0.001	0.46 (0.41–0.51)	0.001	0.47 (0.4–0.53)	0.001
2010–2021	−0.02 (−0.05 to 0.02)	0.33700	−0.05 (−0.08 to −0.02)	0.001	−0.06 (−0.09 to −0.02)	0.001
1990–2021	−0.08 (−0.11 to −0.05)	0.001	−0.05 (‐0.07 to −0.03)	0.001	−0.05 (−0.08 to −0.02)	0.001

*Note:* Data in parentheses are 95% confidence intervals for AAPCs.

Abbreviations: AAPC = average annual percentage change and YLDs = years lived with disability.

FIGURE 2Global map of neck pain incidence, prevalence, and YLDs in 2021. (A) Global map of 2021 incidence of global neck pain (per 100,000 population) in adolescents and young adults aged 10–24 years. (B) Global map of 2021 prevalence of global neck pain (per 100,000 population) in adolescents and young adults aged 10–24 years. (C) Global map of 2021 YLDs of global neck pain (per 100,000 population) in adolescents and young adults aged 10–24 years. YLDs = years lived with disability.

**Figure:**
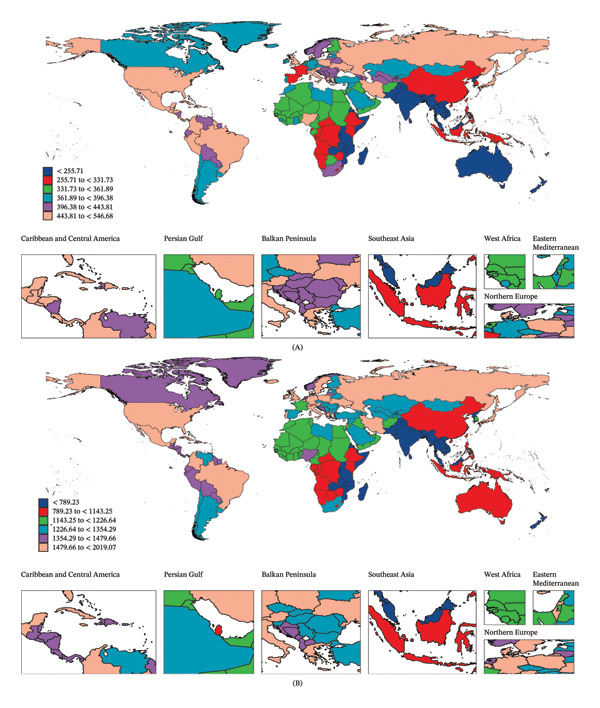

**Figure:**
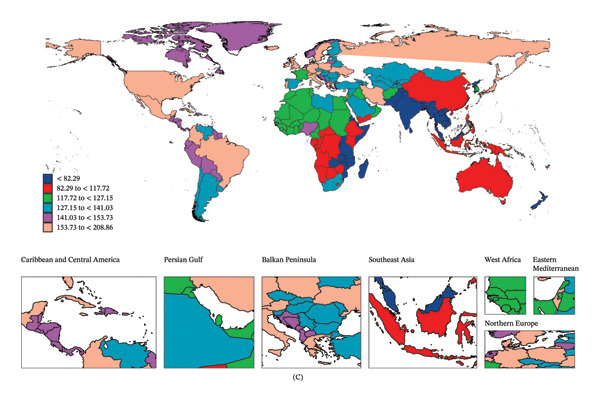

FIGURE 3Global map of average annual percentage changes in incidence, prevalence, and YLDs of neck pain from 1990 to 2021. (A) Global map of AAPC in incidence of neck pain from 1990 to 2021. (B) Global map of AAPC in prevalence of neck pain from 1990 to 2021. (C) Global map of AAPC in YLDs of neck pain from 1990 to 2021. AAPC = average annual percentage change and YLDs = years lived with disability.

**Figure:**
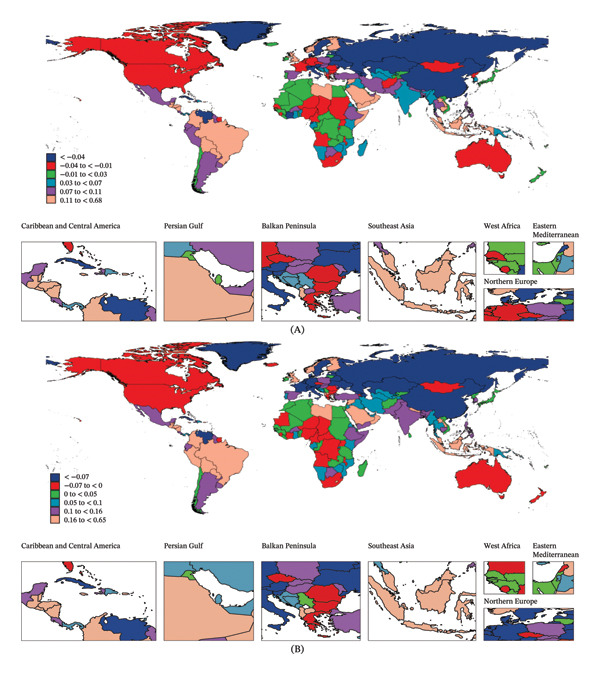

**Figure:**
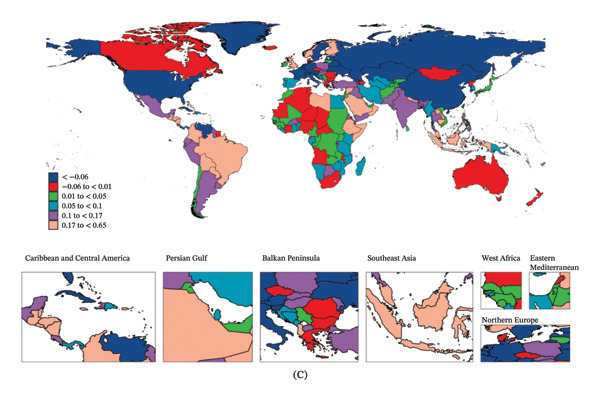

The prevalence decreased from 1990 to 1999 (AAPC –0.5 [95% CI: −0.54 to −0.47], *p* = 0.001), followed by an increase from 2000 to 2009 (AAPC 0.46 [95% CI: 0.41–0.51], *p* = 0.001) then continued to decline from 2010 to 2021, albeit at a slower rate (AAPC –0.05 [–0.08 to −0.02], *p* = 0.001), as presented in Table 1 and Figure 1B. Overall, the age‐standardized prevalence rate of neck pain in 2021 (1019 [95% UI: 549–1742]) was lower than that in 1990 (1029 [95% UI: 556–1755]) per 100,000, indicating an AAPC of −0.05 (95% CI: −0.07 to −0.03, *p* = 0.001; Table 2). The prevalence of neck pain across 204 countries and regions is depicted in Figure 2B. The United States, Russian Federation, Brazil, etc. have higher prevalence rates (Figure 2B). Additionally, the AAPC of prevalence across 204 countries and regions is indicated in Figure 3B. Brazil also showed a high AAPC for incidence (Figure 3B).

**TABLE 2:** The prevalence and YLDs of neck pain and their AAPCs from 1990 to 2021 at the global and regional levels.

	Prevalence						YLDs					
	Cases (*n*), 1990	Age‐standardized rate in 1990 (per 100,000)	Cases (*n*), 2021	Age‐standardized rate in 2021 (per 100,000)	AAPC, 1990–2021	*p* value	Cases (*n*), 1990	Age‐standardized rate in 1990 (per 100,000)	Cases (*n*), 2021	Age‐standardized rate in 2021 (per 100,000)	AAPC, 1990–2021	*p* value
Global	16,092,049 (10,017,822–2,568,388)	1029 (556–1755)	19,349,609 (12,099,446–30,788,326)	1019 (549–1742)	−0.05 (−0.07 to −0.03)	0.001	1,668,979 (875,062–3,006,159)	107 (52–200)	2,005,643 (1,054,190–3,587,510)	106 (51–197)	−0.05 (−0.08 to −0.02)	0.001

*Sex*												
Male	7,041,640 (4,345,119–11,218,998)	889 (476–1381)	8,370,387 (5,178,454–13,464,129)	863 (459–1365)	−0.12 (−0.14 to −0.09)	0.001	734,985 (388,264–1,336,437)	93 (45–200)	874,260 (458,416–1,573,860)	90 (43–197)	−0.11 (−0.14 to −0.09)	0.001
Female	9,050,410 (5,615,576–14,467,958)	1173 (637–1381)	10,979,222 (6,857,295–17,460,392)	1182 (638–1365)	0.01 (−0.02–0.03)	0.638	933,994 (490,914–1,673,252)	121 (59–200)	1,131,384 (599,347–2,021,801)	122 (60–197)	0 (−0.03–0.04)	0.769

*Age group, years*												
10–14	2,656,335 (1,455,274–4,318,237)	496 (272–1381)	3,336,182 (1,835,635–5,413,796)	500 (275–1365)	0.03 (0.01–0.04)	0.001	277,703 (131,311–488,278)	52 (25–200)	348,740 (165,452–617,880)	52 (25–197)	0.03 (0.01–0.05)	0.001
15–19	5,273,779 (2,915,904–8,844,790)	1015 (561–1381)	6,314,842 (3,465,172–10,691,656)	1012 (555–1365)	−0.02 (−0.04 to 0.01)	0.161	548,000 (256,592–1,034,866)	106 (49–200)	655,849 (306,171–1,225,014	105 (49–197)	−0.02 (−0.04 to 0)	0.126
20–24	8,161,935 (4,319,918–14,289,541)	1659 (878–1381)	9,698,585 (5,126,949–16,991,537)	1624 (859–1365)	−0.07 (−0.09 to −0.04)	0.001	843,275 (425,160–1,604,167)	171 (86–200)	1,001,054 (502,943–1,895,792)	168 (84–197)	−0.07 (−0.1 to −0.05)	0.001

*Sociodemographic index*												
High‐middle	3,224,583 (2,002,014–5,172,505)	1090 (591–1852)	2,502,859 (1,542,410–4,006,026)	1088 (582–1846)	−0.09 (−0.12 to −0.06)	0.001	335,883 (175,794–611,897)	114 (56–213)	260,665 (137,079–465,576)	113 (55–211)	−0.09 (−0.12 to −0.05)	0.001
High	2,855,794 (1,785,365–4,515,433)	1400 (766–2377)	2,755,214 (1,733,320–4,324,750)	1432 (788–2422)	0.05 (0.01–0.09)	0.013	296,250 (157,647–525,878)	145 (71–266)	284,538 (151,479–501,774)	148 (72–268)	0.04 (0–0.07)	0.028
Low‐middle	3,096,965 (1,960,461–4,933,847)	878 (474–1511)	4,978,684 (3,128,870–7,943,527)	894 (483–1538)	0.17 (0.13–0.21)	0.001	319,762 (171,065–569,663)	91 (44–170)	515,475 (274,720–916,078)	93 (45–174)	0.18 (0.12–0.25)	0.001
Low	1,394,745 (879,601–2,205,220)	934 (502–1599)	3,446,158 (2,170,464–5,467,635)	958 (515–1640)	0.13 (0.1–0.16)	0.001	143,168 (76,248–254,794)	96 (47–178)	355,976 (186,890–634,811)	99 (48–184)	0.15 (0.13–0.18)	0.001
Middle	5,503,385 (3,378,708–8,807,648)	980 (523–1690)	5,650,003 (3,454,803–9,082,904)	1011 (540–1737)	0.06 (0.01–0.11)	0.013	572,198 (300,136–1,042,011)	102 (49–193)	587,259 (310,642–1,054,034)	105 (51–197)	0.06 (0–0.12)	0.064

*Region*												
East Asia	3,236,128 (1,952,383–5,293,519)	803 (424–1381)	1,924,979 (1,177,616–3,139,984)	793 (422–1365)	−0.33 (−0.45 to −0.21)	0.001	338,702 (180,676–639,133)	84 (40–160)	202,058 (107,782–361,288)	83 (39–155)	−0.32 (−0.45 to −0.2)	0.001
Oceania	16,602 (10,106–26,930)	813 (433–1402)	32,559 (19,782–53,182)	812 (433–1399)	0.06 (0.03–0.08)	0.001	1720 (923–3146)	84 (41–159)	3386 (1796–6271)	84 (39–162)	0.08 (0.01–0.15)	0.035
Central Asia	258,071 (158,611–411,528)	1312 (694–2245)	287,905 (177,025–460,217)	1310 (692–2239)	0 (−0.04 to 0.03)	0.845	26,820 (13,838–47,832)	136 (65–257)	29,925 (15,596–53,710)	136 (66–255)	0 (−0.03–0.04)	0.81
Central Europe	401,015 (248,053–641,814)	1379 (729–2352)	256,225 (157,496–414,127)	1387 (734–2367)	0.09 (0.05–0.13)	0.001	41,733 (21,753–74,282)	144 (68–270)	26,671 (13,749–48,368)	144 (70–275)	0.09 (0.05–0.13)	0.001
Eastern Europe	726,176 (448,233–1,173,857)	1524 (818–2605)	494,187 (307,989–793,212)	1527 (820–2608)	−0.09 (−0.12 to −0.05)	0.001	75,537 (39,333–138,052)	159 (76–300)	51,435 (27,020–93,386)	159 (76–301)	−0.08 (−0.12 to −0.04)	0.001
High‐income Asia Pacific	606,125 (374,453–967,835)	1393 (745–2426)	381,631 (235,317–616,045)	1395 (746–2429)	0.05 (0.02–0.08)	0.001	63,163 (32,752–113,294)	145 (70–276)	39,767 (20,885–70,850)	145 (70–276)	0.05 (−0.01–0.1)	0.099
Australasia	42,525 (26,121–66,520)	864 (464–1492)	50,289 (30,998–78,697)	862 (463–1489)	−0.03 (−0.06 to 0)	0.033	4391 (2319–7879)	89 (42–171)	5189 (2688–9018)	89 (42–168)	−0.02 (−0.1 to 0.06)	0.696
Western Europe	1,291,699 (824,457–2,030,512)	1506 (852–2529)	1,155,132 (730,021–1,821,993)	1570 (871–2648)	0.06 (0–0.12)	0.043	133,750 (71,771–231,636)	156 (78–281)	119,428 (64,118–206,330)	162 (80–294)	0.04 (−0.03–0.12)	0.248
Southern Latin America	170,669 (105,197–270,859)	1296 (700–2222)	204,081 (125,202–327,640)	1294 (699–2218)	0.1 (0.09–0.11)	0.001	17,721 (9340–31,790)	135 (64–250)	21,115 (11,169–38,135)	134 (65–249)	0.1 (0.07–0.12)	0.001
High‐income North America	992,274 (614,853–1,602,084)	1553 (842–2634)	1,142,422 (709,384–1,835,382)	1553 (842–2635)	−0.06 (−0.17 to 0.04)	0.247	102,630 (54,141–185,584)	161 (78–297)	117,249 (61,482–211,014)	159 (77–294)	−0.08 (−0.14 to −0.02)	0.006
Caribbean	153,171 (92,950–245,916)	1406 (749–2408)	163,679 (99,314–263,165)	1403 (748–2404)	0.03 (0.01–0.05)	0.008	15,820 (8255–28,347)	145 (69–272)	16,914 (8851–30,628)	145 (69–269)	0.03 (0.01–0.05)	0.004
Andean Latin America	168,991 (103,678–269,194)	1406 (749–2408)	248,958 (151,348–400,261)	1400 (746–2401)	0.16 (0.15–0.18)	0.001	17,525 (9117–31,044)	146 (69–271)	25,803 (13,517–46,322)	145 (69–272)	0.16 (0.13–0.18)	0.001
Central Latin America	824,332 (510,490–1,311,101)	1553 (834–2671)	1,027,612 (625,797–1,628,189)	1542 (828–2655)	0.13 (0.12–0.13)	0.001	85,657 (45,072–151,266)	161 (77–299)	106,745 (56,090–188,715)	160 (77–298)	0.13 (0.11–0.14)	0.001
Tropical Latin America	764,872 (464,839–1,253,719)	1625 (866–2830)	862,576 (519,619–1,429,346)	1619 (864–2818)	0.21 (0.16–0.26)	0.001	78,919 (41,798–144,115)	168 (81–323)	88,847 (46,638–163,470)	167 (80–321)	0.2 (0.15–0.26)	0.001
North Africa and Middle East	1,330,895 (835,207–2,139,263)	1259 (682–2136)	1,995,750 (1,251,404–3,237,386)	1234 (670–2093)	0.02 (0–0.04)	0.082	137,504 (73,525–244,229)	130 (64–241)	206,302 (110,783–367,598)	128 (63–237)	0.02 (0–0.05)	0.058
South Asia	2,252,543 (1,416,532–3,588,330)	685 (367–1187)	3,671,706 (2,293,990–5,903,172)	687 (368–1190)	0.12 (−0.04–0.28)	0.143	232,330 (123,016–408,533)	71 (34–133)	380,050 (200,997–677,946)	71 (34–133)	0.14 (−0.02–0.29)	0.092
Central sub‐Saharan Africa	183,283 (115,511–289,880)	1094 (585–1880)	473,395 (298,028–748,742)	1094 (584–1880)	−0.02 (−0.02 to −0.01)	0.001	18,686 (9974–33,647)	112 (53–208)	48,723 (25,709–86,178)	113 (53–211)	0.01 (−0.01–0.03)	0.281
Eastern sub‐Saharan Africa	490,164 (304,332–768,606)	826 (444–1412)	1,169,573 (726,161–1,843,413)	824 (443–1408)	0.06 (0.02–0.11)	0.009	50,478 (26,671–89,327)	85 (41–158)	121,168 (62,901–216,385)	85 (41–159)	0.08 (0.04–0.12)	0.001
Southern sub‐Saharan Africa	211,310 (130,501–332,471)	1259 (681–2196)	270,156 (166,361–425,430)	1242 (671–2161)	0 (−0.02–0.03)	0.932	21,869 (11,567–38,731)	130 (63–242)	27,922 (14,762–49,451)	128 (63–239)	0 (−0.02 to 0.02)	0.847
Western sub‐Saharan Africa	779,821 (492,841–1,229,791)	1355 (732–2321)	2,094,672 (1,323,935–3,302,848)	1351 (730–2314)	−0.01 (−0.02 to 0)	0.003	80,066 (42,197–142,227)	139 (68–260)	216,511 (114,918–383,274)	140 (68–262)	0.01 (0–0.02)	0.12
Southeast Asia	1,191,383 (736,995–1,905,045)	814 (431–1415)	1,442,124 (882,981–2,323,100)	823 (435–1432)	0.16 (0.16–0.16)	0.001	123,959 (65,217–222,226)	85 (40–160)	150,433 (79,493–274,305)	86 (41–163)	0.17 (0.16–0.18)	0.001

*Note:* Data in parentheses are 95% uncertainty intervals for cases, prevalence, and YLDs, and 95% confidence intervals for AAPCs.

Abbreviations: AAPC = average annual percentage change, YLDs = years lived with disability.

The YLDs decreased from 1990 to 1999 (AAPC –0.5 [95% CI: −0.54 to −0.46], *p* = 0.001), followed by an increase from 2000 to 2009 (AAPC 0.47 [95% CI: 0.4–0.53], *p* = 0.001), and then continued to decline from 2010 to 2021, albeit at a slower rate (AAPC –0.06 [–0.09 to −0.02], *p* = 0.001), as presented in Table 1 and Figure 1C. Overall, the age‐standardized YLDs rate of neck pain in 2021 (106 [95% UI: 51–197]) was lower than the incidence rate in 1990 (107 [95% UI: 52–200] per 100,000, with an AAPC of −0.05 [95% CI: −0.08 to −0.02], *p* = 0.001; Table 2). The YLDs of neck pain across 204 countries and regions are depicted in Figure 2C. The United States, Russian Federation, Brazil, etc. have higher prevalence rates (Figure 2B). Additionally, the AAPC of YLDs in 204 countries and regions is shown in Figure 3C. Brazil also showed a high AAPC for incidence (Figure 3B).

### 3.2. Global Trends by Sex

Global neck pain incidence rates decreased for males from 275 (95% UI: 120–487) in 1990 to 263 (95% UI: 116–495) per 100,000 in 2021, with an AAPC of −0.15 (95% CI:–0.17 to −0.12, *p* = 0.001; Supporting Table 1 and Supporting Figure 1). Prevalence and YLDs showed similar trends: prevalence decreased from 889 (95% UI: 476–1381) to 863 (95% UI: 459–1365) per 100,000, and YLDs decreased from 93 (95% UI: 45–200) to 90 (95% UI: 43–197) per 100,000 (Supporting Table 1 and Supporting Figures 2 and 3). For females, the incidence, prevalence, and YLDs showed numerical increases from 1990 to 2021, but these changes did not reach statistical significance (Supporting Table 1 and Supporting Figures 4–6). Supporting Figures 7–12 illustrate the incidence, prevalence, and YLDs of neck pain by sex across 204 countries and regions.

### 3.3. Global Trends by Age

Globally, the most significant reduction in incidence from 1990 to 2021 occurred among young adults aged 20–24 years (declining from 447 [95% UI: 169–487] in 1990 to 432 [95% UI: 168–495] per 100,000 in 2021, with an AAPC of −0.11 [95% CI: −0.13 to −0.08], *p* = 0.001), as indicated in Supporting Table 1 and Supporting Figures 13–15. During the same period, the prevalence (from 1659 [95% UI: 878–1381] to 1624 [95% UI: 859–1365] per 100,000, with an AAPC of −0.07 [95% CI: −0.09 to −0.04], *p* = 0.001) and YLDs (from 171 [95% UI: 86–200] to 168 [95% UI: 84–197] per 100,000, with an AAPC of −0.07 [95% CI: −0.1 to −0.05], *p* = 0.001) of neck pain in young adults aged 20–24 years also declined most notably (Table 2 and Supporting Figures 16–21). Only those aged 10–14 years experienced a significant increase in prevalence (from 496 [95% UI: 272–1381] to 500 [95% UI: 275–1365] per 100,000, with an AAPC of 0.03 [95% CI: 0.01–0.04], *p* = 0.001) of neck pain. More details are provided in Table 2 and Supporting Table 1. The incidence, prevalence, and YLDs of neck pain across 204 countries and regions by age are shown in Supporting Figures 22–30.

### 3.4. Global Trends by SDI

The sole decrease in neck pain incidence among SDI quintiles was high‐middle SDI (from 339 [95% UI: 148–615] to 334 [95% UI: 147–599] per 100,000, with an AAPC of −0.11 [95% CI: −0.14 to −0.08], *p* = 0.001). Moreover, prevalence and YLDs decreased in countries in the high‐middle‐SDI quintile, from 1090 [95% UI: 591–1852] in 1990 to 1088 [95% UI: 582–1846] in 2021 and from 114 [95% UI: 56–213] in 1990 to 113 [95% UI: 55–211] per 100,000 in 2021, respectively.

Countries in the low‐SDI quintile, low‐middle‐SDI quintile, and high‐SDI quintile showed increased incidence, prevalence, and YLDs of neck pain from 1990 to 2021 (Table 2 and Supporting Table 1). Notably, in 2021, the high‐SDI‐quintile countries exhibited the highest neck pain prevalence (1432 [95% UI: 788–2422]) and YLDs (148 [95% UI: 72–268]; Table 2).

### 3.5. Regional Trends

Among the 21 GBD regions, Western Europe experienced the most significant increase in the incidence of neck pain from 1990 to 2021, rising from 385 (95% UI: 177–693) to 402 (95% UI: 184–729) per 100,000, with an AAPC of 0.09 (95% CI: 0.03–0.15, *p* = 0.003). Additionally, the largest increase in neck pain prevalence was observed in Western Europe (from 1506 [95% UI: 852–2529] to 1570 [95% UI: 871–2648] per 100,000, with an AAPC of 0.06 [95% CI: 0–0.12], *p* = 0.043; Table 2). The region with the highest age‐standardized incidence rate of neck pain in 2021 was Tropical Latin America (523 [95% UI: 230–953] per 100,000). This region also recorded the most significant prevalence at 1619 [95% UI: 864–2818] per 100,000 and YLDs of 167 [95% UI: 80–321] per 100,000 (Table 2). The AAPC for 204 countries and regions is indicated in Figure 3.

### 3.6. Decomposition Analysis of Change in YLDs

**FIGURE 4:**
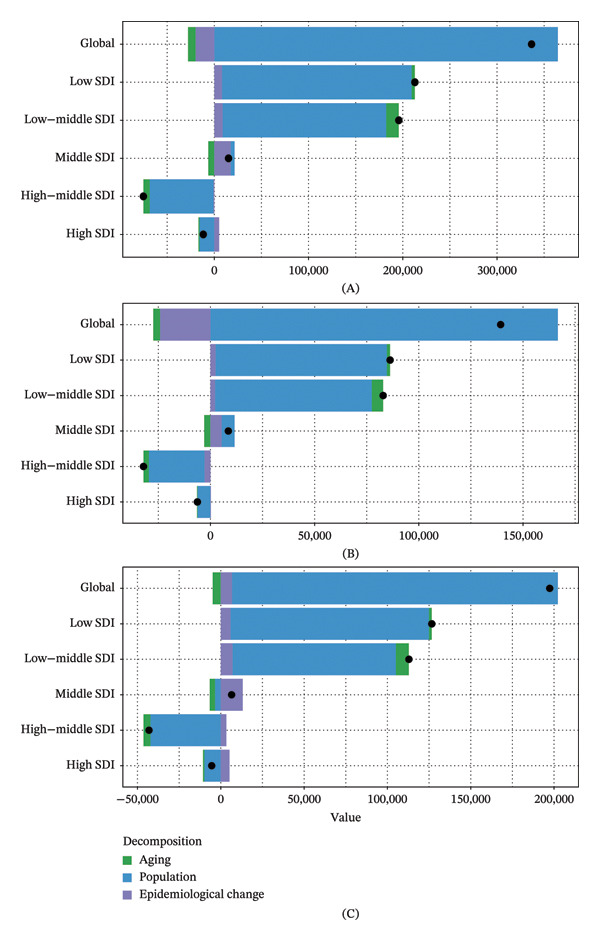
Change in YLDs of preterm birth decomposed by three population‐level determinants: aging, population, and epidemiological change at the global level and various regions. (A) Decomposition analysis of change in YLDs in both genders. (B) Decomposition analysis of change in YLDs in male. (C) Decomposition analysis of change in YLDs in female. The black dots indicate the total value of change attributable to all three components. SDI, sociodemographic index.

The decomposition analysis of changes in YLDs due to three population‐level determinants (aging, demographic and epidemiological changes) across the five SDI regions is shown in Figure 4 and Supporting Table 2. Globally, population growth accounted for 108.3% of the change in disability, followed by epidemiological changes (−5.86%) and aging (−2.44%). Among the five SDI regions, the high‐SDI region was most affected by these determinants: population change contributed 133.71%, epidemiological changes −43.41%, and aging 9.71% (Supporting Table 2). Aging was least affected by low‐SDI regions (1.61%), population was least affected by middle‐SDI regions (27.38%), and epidemiological change was least affected by high‐middle‐SDI regions (1.09%). The contribution of population to YLDs was positive in all regions (Figure 4 and Supporting Table 2). The contribution of aging to YLDs was positive in all regions except for the middle‐SDI regions (Figure 4 and Supporting Table 2). However, the contribution of aging to YLDs was positive in all regions except for the high‐SDI regions (Figure 4 and Supporting Table 2). Gender affected the decomposition analysis results only for the three regions of middle‐SDI, high‐middle‐SDI, and high‐SDI (Figure 4 and Supporting Table 2).

## 4. Discussion

### 4.1. Summary of Key Results

This study provides a comprehensive assessment of the incidence, prevalence, and YLDs related to neck pain among adolescents and young adults aged 10–24 years in 204 countries and regions from 1990 to 2021 at the global, regional, and national levels. Our findings reveal three novel insights. First, although the global age‐standardized prevalence declined slightly (AAPC –0.05), the absolute number of cases and YLDs continues to rise, and notably, adolescents aged 10–14 years are the only age subgroup with a significant increasing trend (AAPC 0.03)—an emerging concern in early adolescence. Second, the first decomposition analysis of YLDs for this population shows that population growth accounts for 108.3% of the global increase in YLDs, while favorable epidemiological changes only partially offset this effect. Third, by extending data through 2021, we demonstrate that the COVID‐19 pandemic did not reverse the long‐term declining trend. Females consistently experience higher rates than males across all metrics. Regionally, Tropical Latin America had the highest burden in 2021, and high‐SDI countries exhibited the highest prevalence and YLDs.

### 4.2. Global Trends and Comparison With Prior Studies

GBD 2021 report identified neck pain as the 15th most prevalent condition among 371 diseases [4]. We found that the AAPC of incidence decreased between 1990 and 2021, but the overall number of cases continued to increase. Additionally, the total YLDs increased by 21.2% from 1990 to 2021, imposing a significant burden on patients and their families. Neck pain significantly impacts the educational achievement and well‐being of young adults and teenagers. It is concerning that strategies for effective prevention and management have not yet been put into practice [17]. Furthermore, our comprehension of the different determinants and risk factors for neck pain remains lacking [4, 18]. The lack of leadership in implementing specific musculoskeletal health strategies at both national and global levels may result in policymakers, funders, and primary care practitioners giving minimal priority to musculoskeletal conditions [2, 19]. This may be because neck pain is not a uniform condition but a symptomatic state linked to various underlying factors, with each individual experiencing pain uniquely. Various factors can lead to the onset of neck pain, such as psychological issues, genetic predispositions, disruptions in sleep, tobacco use, excess body weight, and a lack of physical activity [20–23].

A recent study by Fu et al. [10] also used GBD data to examine neck pain prevalence trends among individuals aged 10–24 years. Both studies observed a globally stable or slightly decreasing trend in age‐standardized prevalence, consistently higher prevalence in females than in males, and the highest burden among those aged 20–24 years, with a clear age gradient. Regionally, both reported high prevalence in Western Europe and high‐income North America and lower rates in sub‐Saharan Africa. However, our study extends this prior work in several ways. We analyze data through 2021, covering the COVID‐19 pandemic period, and confirm that the pandemic did not reverse the long‐term declining trend. We go beyond prevalence to report incidence and YLDs, providing a more complete picture of the disease burden. Additionally, we applied joinpoint regression and decomposition analysis, which revealed nonmonotonic trends (an increase during 2000–2009 followed by resumed decline) and quantified that population growth was the dominant positive driver of YLDs, partially offset by favorable epidemiological changes. These insights are not available in their study. Thus, while Fu et al. established an important baseline, our study provides a more current, comprehensive, and analytically rigorous assessment.

### 4.3. Consistency and Validation of Core Findings

Our core findings align closely with those reported by the GBD 2021 Neck Pain Collaborators [2] and by Fu et al. [10]. This convergence across different GBD data versions (2019 vs. 2021) and analytical methods (EAPC vs. joinpoint regression) validates the robustness of these patterns. The standardized GBD methodology—including DisMod‐MR 2.1 modeling, uniform SDI construction, and 1000‐draw UIs—ensures that cross‐study comparisons are meaningful. Thus, the observed demographic and socioeconomic disparities reflect genuine, replicable features of the global neck pain burden in this population.

### 4.4. Global Trends by Sex

Greater attention to females is essential, as their incidence rate is 1.31 times that of males. Despite the various factors influencing the dimensions of the cervical spine and paraspinal muscles, females are generally smaller than males [24]. This size disparity may play a role in the differences between sexes regarding the occurrence of post‐traumatic neck pain, such as that resulting from motor vehicle accidents. Studies suggest biological (genetic) factors may also contribute to the differences in pain perception between sexes [25]. The specific reasons for this disparity warrant further investigation.

### 4.5. Global Trends by Age

While only the age group of 10–14 years displayed an upward trend in the AAPC of incidence, those aged 20–24 still exhibited the highest prevalence and YLDs. Neck pain has become increasingly common in recent years, particularly among young individuals [2, 26, 27]. The rising prevalence specifically in the 10–14 years subgroup is concerning. Early adoption of digital technology—smartphones, tablets, and laptops—promotes forward head posture and sustained cervical flexion, increasing mechanical stress on the developing cervical spine [28–30]. Concurrent declines in physical activity and outdoor play may reduce musculoskeletal resilience. Although the COVID‐19 pandemic did not reverse the overall trend, the shift to remote learning may have exacerbated sedentary behavior and screen exposure in this age group. Thus, promoting ergonomic habits, regular physical activity, and early postural education is crucial for preventing neck pain in early adolescence.

Furthermore, injuries sustained during sports or in the workplace are linked to neck pain, particularly in activities such as racing, wrestling, and ice hockey, indicating the highest rates of occurrence [31]. Although office workers and those using computers are at a higher risk of neck and shoulder discomfort, the main job‐related elements linked to neck pain are low job satisfaction and a lack of perceived support in their roles [32]. Consequently, providing care for adolescents and young adults is crucial, and parents and teachers should offer them greater emotional support and recognition.

### 4.6. Regional Trends

From the regional perspective, high‐ and high‐middle‐income areas exhibit higher prevalence rates and YLDs. However, caution is needed when interpreting the lower prevalence rates observed in low‐SDI regions. These lower figures may not represent a truly lower incidence but rather a “diagnostic shadow” caused by limited healthcare infrastructure, under‐reporting, and sparse national registries. The GBD framework relies on statistical extrapolation and modeling to fill data gaps in such settings, which may lead to underestimation of the true burden. Consequently, the observed regional differences likely reflect surveillance disparities in addition to genuine variations in environmental or lifestyle factors. The increase in YLDs might be associated with the growing reliance on technological gadgets, such as computers, laptops, and smartphones. Although increased sedentary lifestyles during the COVID‐19 pandemic might contribute to more significant neck pain, our findings did not reflect this trend. Similar results were observed in another study [2]. This issue is crucial as it may influence the availability of medical practitioners, reimbursement insurance, diagnostic testing, and subsequent treatment options, which do not always align with current best evidence practices. A study in Rio de Janeiro observed no correlation between text neck and neck pain in young individuals aged 18–21 years [33]. Consequently, we recommend that future research elucidate the relationship between income and neck pain and identify the associated risks to enhance prevention strategies.

### 4.7. Strengths and Limitations

This study has several important strengths, including the use of the most recent GBD 2021 data, which extends the analysis through the COVID‐19 pandemic period, and the comprehensive assessment of incidence, prevalence, and YLDs rather than prevalence alone. The application of joinpoint regression and decomposition analysis provides deeper insights into nonmonotonic trend changes and the drivers of YLDs, with population growth identified as the dominant contributor. However, several limitations must be acknowledged. First, the quality and availability of GBD data vary considerably across countries, with particularly sparse primary data from economically disadvantaged regions. Despite the GBD’s efforts to fill gaps through statistical modeling, the true burden in low‐SDI areas may be underestimated—a “diagnostic shadow” due to limited healthcare infrastructure and under‐reporting. Second, the GBD case definition of neck pain (discomfort lasting at least one day) does not distinguish between traumatic and non‐traumatic causes. This distinction is important because the etiology, risk factors, and clinical course of traumatic neck pain (e.g., whiplash from sports or accidents) differ substantially from those of nontraumatic degenerative or posture‐related pain. Third, the same definition does not differentiate between acute, recurrent, and chronic neck pain. Acute episodes may resolve quickly and have low impact, whereas chronic or recurrent pain contributes disproportionately to disability and healthcare utilization. Consequently, our prevalence and YLDs estimates reflect any neck pain lasting at least one day, regardless of chronicity or recurrence. Fourth, the decomposition analysis assumes that the contributions of population growth, aging, and epidemiological changes are additive and independent, which may not fully capture complex interactions. Similarly, joinpoint regression assumes that trend changes occur at discrete time points, and the permutation test for selecting the optimal number of joinpoints is subject to multiple comparison considerations. Finally, there is an urgent need to standardize neck pain data collection globally and to encourage primary data collection in low‐income countries to improve the accuracy and comparability of future burden estimates.

## 5. Conclusion

Between 1990 and 2021, a decrease in the global prevalence of neck pain among adolescents and young adults between the ages of 10 and 24 years was observed. However, a notable increase in prevalence was observed in adolescents between the ages of 10–14 years. Besides, the prevalence rate is considerably greater in females than in males, and it is also notably higher in young adults than in adolescents. Therefore, urgent interventions are needed to prevent neck pain in adolescents and young adults.

## Author Contributions

Fei‐Long Wei, Yi‐Fang Yuan, and Shu Qian designed the study. Shu Qian, Sheng‐Jie Guo, Yang Song, Hao‐Ran Gao, and Fei‐Long Wei analyzed the data and did the statistical analyses. Shu Qian, Yi‐Fang Yuan, and Fei‐Long Wei drafted the initial manuscript.

## Funding

The authors have nothing to report.

## Disclosure

All authors reviewed the draft manuscript and endorsed the final version. All authors had complete access to the data and take responsibility for submitting it for publication.

## Ethics Statement

The authors have nothing to report.

## Conflicts of Interest

The authors declare no conflicts of interest.

## Supporting Information

Additional supporting information can be found online in the Supporting Information section.

## Supporting information


**Supporting Information** This file contains Supporting Figures 1–30 (joinpoint regression trends and global maps of incidence, prevalence, and YLDs by sex and age group) and Supporting Tables 1–2 (age‐standardized incidence rates with AAPCs, and decomposition analysis of YLDs changes) for neck pain in adolescents and young adults aged 10–24 years from 1990 to 2021.

## Data Availability

The data that support the findings of this study are available in the Supporting Information of this article.
