# Supplementary material for: Global, Regional, and National Burdens of Neck Pain in Adolescents and Young Adults Aged 10–24 Years From 1990 to 2021: A Population‐Based Study
Source: Pain Res Manag. 2026 Jul 23;2026:3439180. doi: 10.1155/prm/3439180 (PMC13396697; doi:10.1155/prm/3439180)
Supplement: Supplementary file 1 — Supporting Information This file contains Supporting Figures 1–30 (joinpoint regression trends and global maps of incidence, prevalence, and YLDs by sex and age group) and Supporting Tables 1–2 (age‐standardized incidence rates with AAPCs, and decomposition analysis of YLDs changes) for neck pain in adolescents and young adults aged 10–24 years from 1990 to 2021. [file PRM-2026-3439180-s001.doc]

**Supplementary Materials**

**Supplement files**

**1. Figures** (Supplement Figures 1-30)

Supplement Figure 1. Joinpoint regression analysis of global neck pain incidence in male (in adolescents and young adults aged 10-24 years from 1990 to 2021).

Supplement Figure 2. Joinpoint regression analysis of global neck pain prevalence in male (in adolescents and young adults aged 10-24 years from 1990 to 2021).

Supplement Figure 3. Joinpoint regression analysis of global neck pain YLDs in male (in adolescents and young adults aged 10-24 years from 1990 to 2021).

Supplement Figure 4. Joinpoint regression analysis of global neck pain incidence in female (in adolescents and young adults aged 10-24 years from 1990 to 2021).

Supplement Figure 5. Joinpoint regression analysis of global neck pain prevalence in female (in adolescents and young adults aged 10-24 years from 1990 to 2021).

Supplement Figure 6. Joinpoint regression analysis of global neck pain YLDs in female (in adolescents and young adults aged 10-24 years from 1990 to 2021).

Supplement Figure 7. Global map of 2021 incidence of neck pain in male (per 100,000 population) in adolescents and young adults aged 10-24 years from 1990 to 2021.

Supplement Figure 8. Global map of 2021 prevalence of neck pain in male (per 100,000 population) in adolescents and young adults aged 10-24 years from 1990 to 2021.

Supplement Figure 9. Global map of 2021 YLDs of neck pain in male (per 100,000 population) in adolescents and young adults aged 10-24 years from 1990 to 2021.

Supplement Figure 10. Global map of 2021 incidence of neck pain in female (per 100,000 population) in adolescents and young adults aged 10-24 years from 1990 to 2021.

Supplement Figure 11. Global map of 2021 prevalence of neck pain in female (per 100,000 population) in adolescents and young adults aged 10-24 years from 1990 to 2021.

Supplement Figure 12. Global map of 2021 prevalence of neck pain in female (per 100,000 population) in adolescents and young adults aged 10-24 years from 1990 to 2021.

Supplement Figure 13. Joinpoint regression analysis of global neck pain incidence in adolescents and young adults aged 10-14 years from 1990 to 2021.

Supplement Figure 14. Joinpoint regression analysis of global neck pain incidence in adolescents and young adults aged 15-19 years from 1990 to 2021.

Supplement Figure 15. Joinpoint regression analysis of global neck pain incidence in adolescents and young adults aged 20-24 years from 1990 to 2021.

Supplement Figure 16. Joinpoint regression analysis of global neck pain prevalence in adolescents and young adults aged 10-14 years from 1990 to 2021.

Supplement Figure 17. Joinpoint regression analysis of global neck pain prevalence in adolescents and young adults aged 15-19 years from 1990 to 2021.

Supplement Figure 18. Joinpoint regression analysis of global neck pain prevalence in adolescents and young adults aged 20-24 years from 1990 to 2021.

Supplement Figure 19. Joinpoint regression analysis of global neck pain YLDs in adolescents and young adults aged 10-14 years from 1990 to 2021.

Supplement Figure 20. Joinpoint regression analysis of global neck pain YLDs in adolescents and young adults aged 15-19 years from 1990 to 2021.

Supplement Figure 21. Joinpoint regression analysis of global neck pain YLDs in adolescents and young adults aged 20-24 years from 1990 to 2021.

Supplement Figure 22. Global map of 2021 incidence of global neck pain incidence (per 100,000 population) in adolescents and young adults aged 10-14 years.

Supplement Figure 23. Global map of 2021 incidence of global neck pain incidence (per 100,000 population) in adolescents and young adults aged 15-19 years.

Supplement Figure 24. Global map of 2021 incidence of global neck pain incidence (per 100,000 population) in adolescents and young adults aged 20-24 years.

Supplement Figure 25. Global map of 2021 incidence of global neck pain prevalence (per 100,000 population) in adolescents and young adults aged 10-14 years.

Supplement Figure 26. Global map of 2021 incidence of global neck pain prevalence (per 100,000 population) in adolescents and young adults aged 15-19 years.

Supplement Figure 27. Global map of 2021 incidence of global neck pain prevalence (per 100,000 population) in adolescents and young adults aged 20-24 years.

Supplement Figure 28. Global map of 2021 incidence of global neck pain YLDs (per 100,000 population) in adolescents and young adults aged 10-14 years.

Supplement Figure 29. Global map of 2021 incidence of global neck pain YLDs (per 100,000 population) in adolescents and young adults aged 15-19 years.

Supplement Figure 30. Global map of 2021 incidence of global neck pain YLDs (per 100,000 population) in adolescents and young adults aged 20-24 years.

**Table**

Supplementary Table 1: The Incidence of neck pain and their AAPCs from 1990 to 2021 at the global and regional levels.

Supplementary Table 2: Decomposition analysis of change in YLDs.

Supplement Figure 1. Joinpoint regression analysis of global neck pain incidence in male in adolescents and young adults aged 10-24 years from 1990 to 2021.


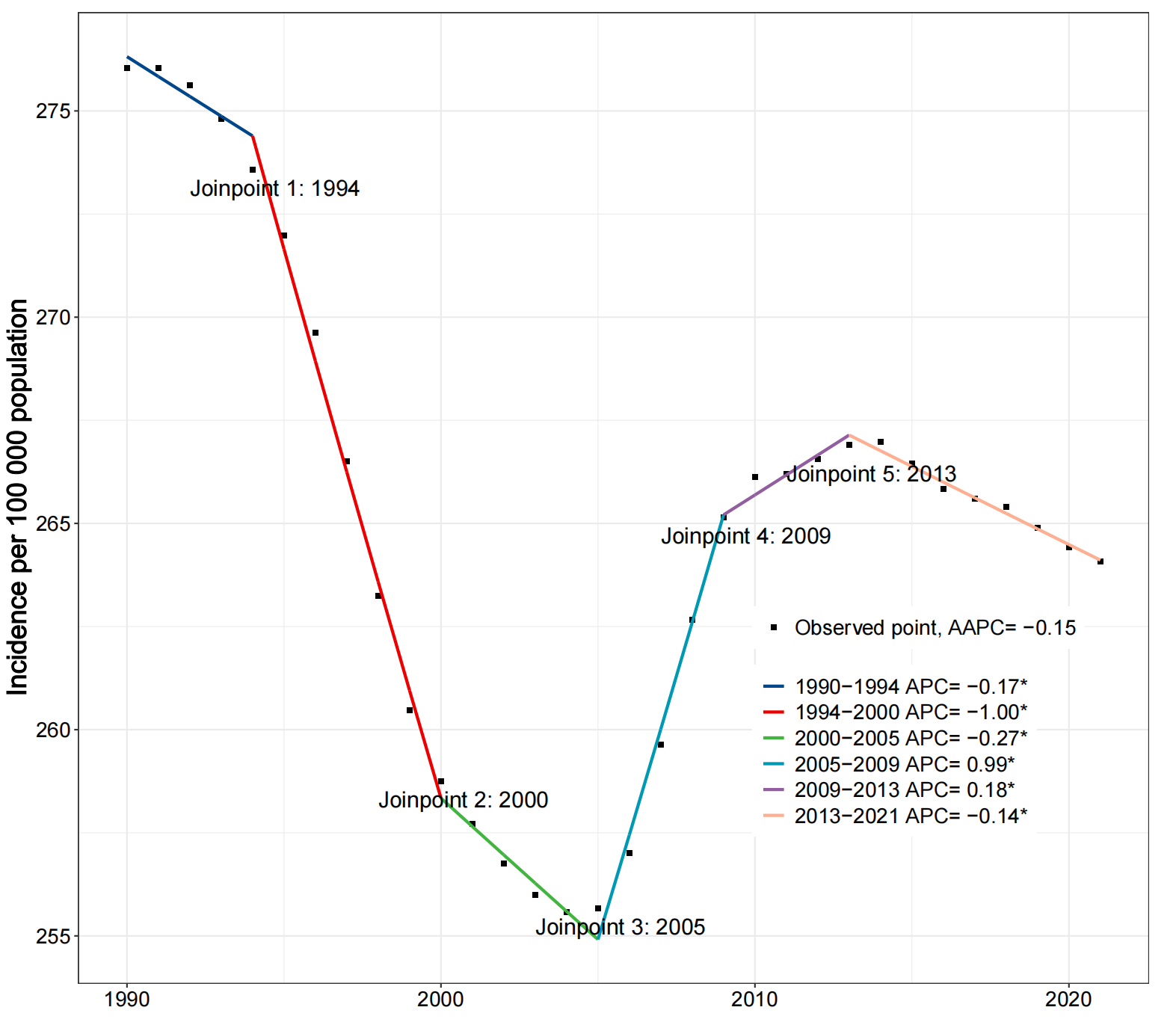


Supplement Figure 2. Joinpoint regression analysis of global neck pain prevalence in male in adolescents and young adults aged 10-24 years from 1990 to 2021.


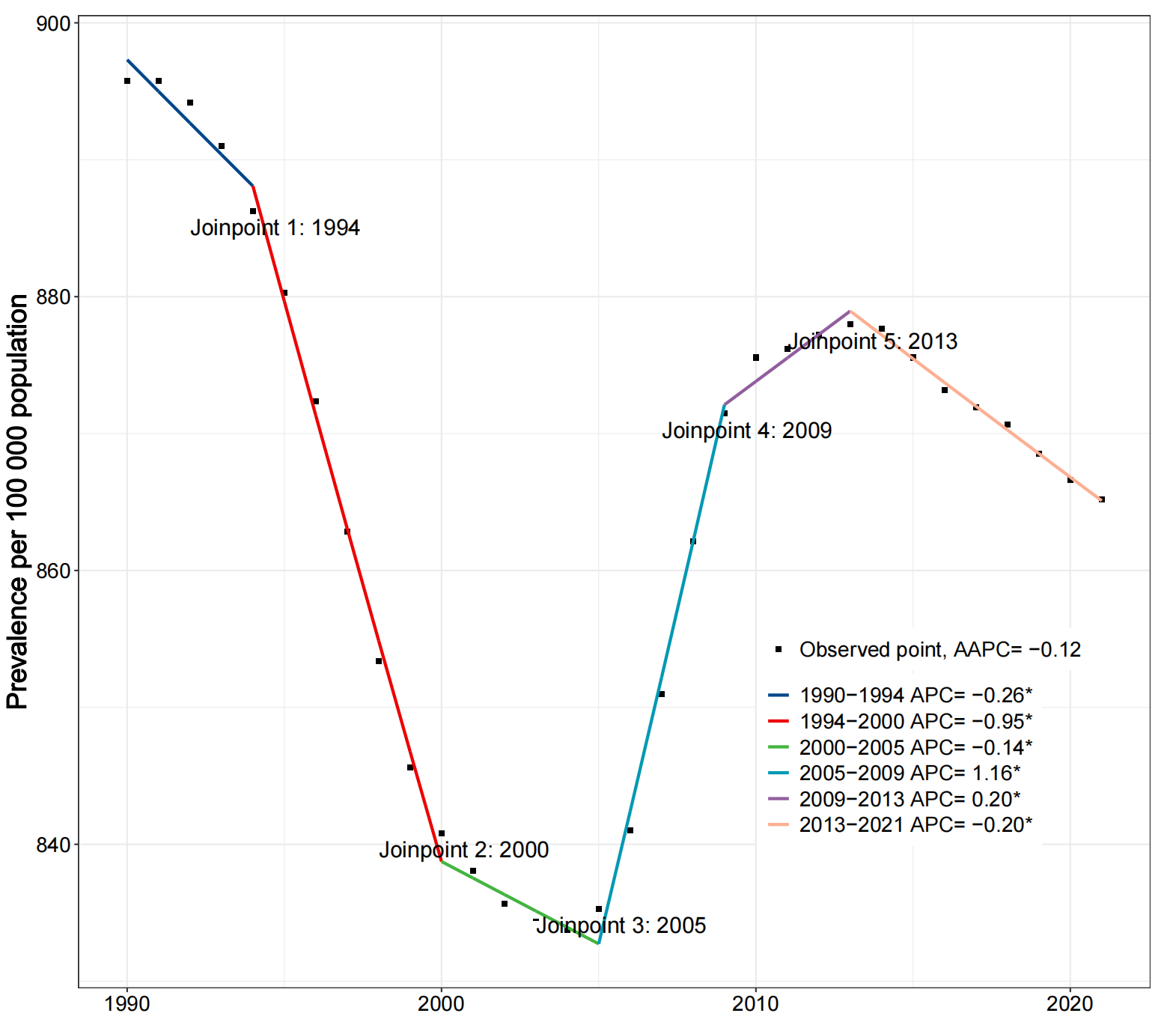


Supplement Figure 3. Joinpoint regression analysis of global neck pain YLDs in male in adolescents and young adults aged 10-24 years from 1990 to 2021.


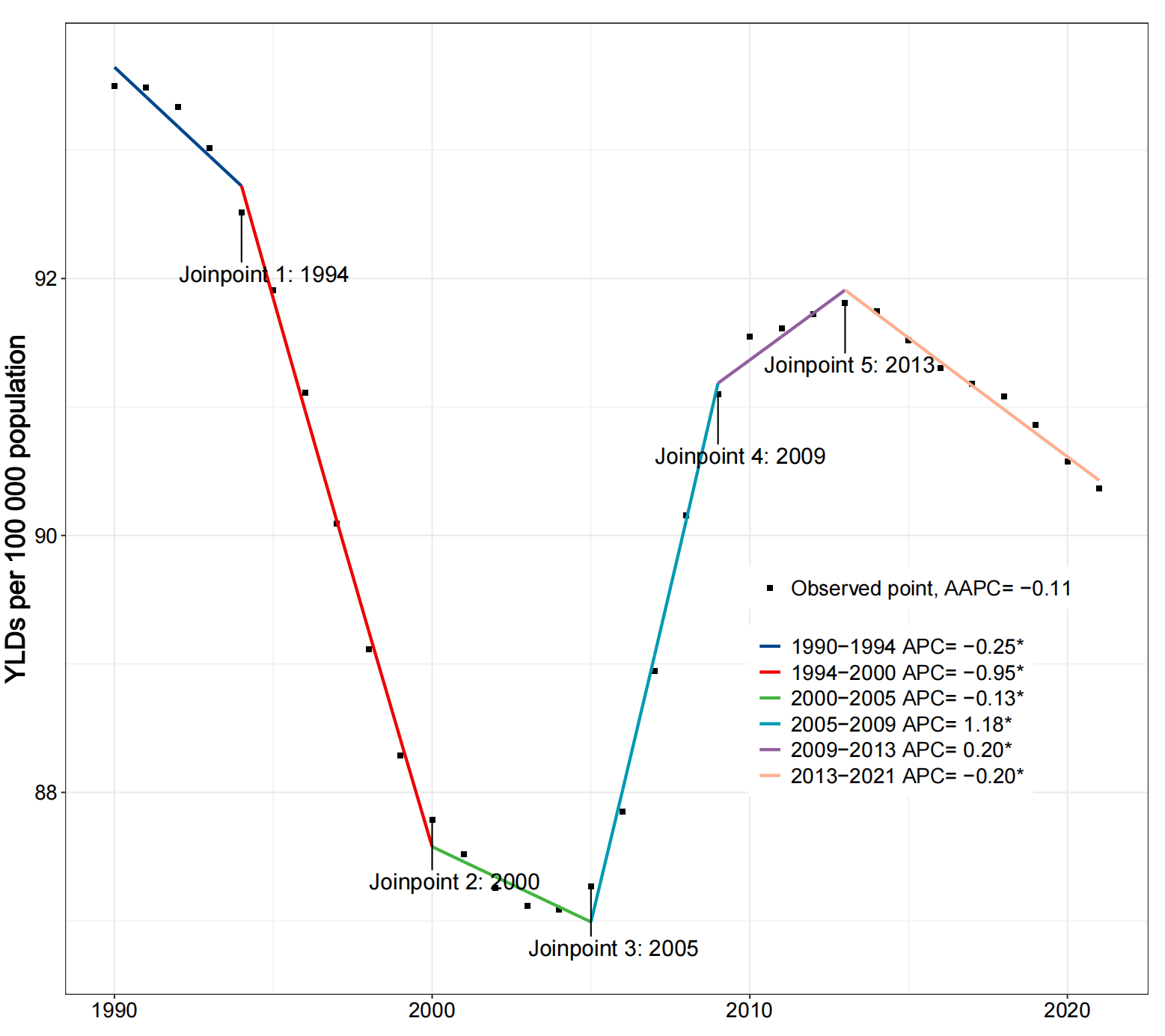


Supplement Figure 4. Joinpoint regression analysis of global neck pain incidence in female in adolescents and young adults aged 10-24 years from 1990 to 2021.


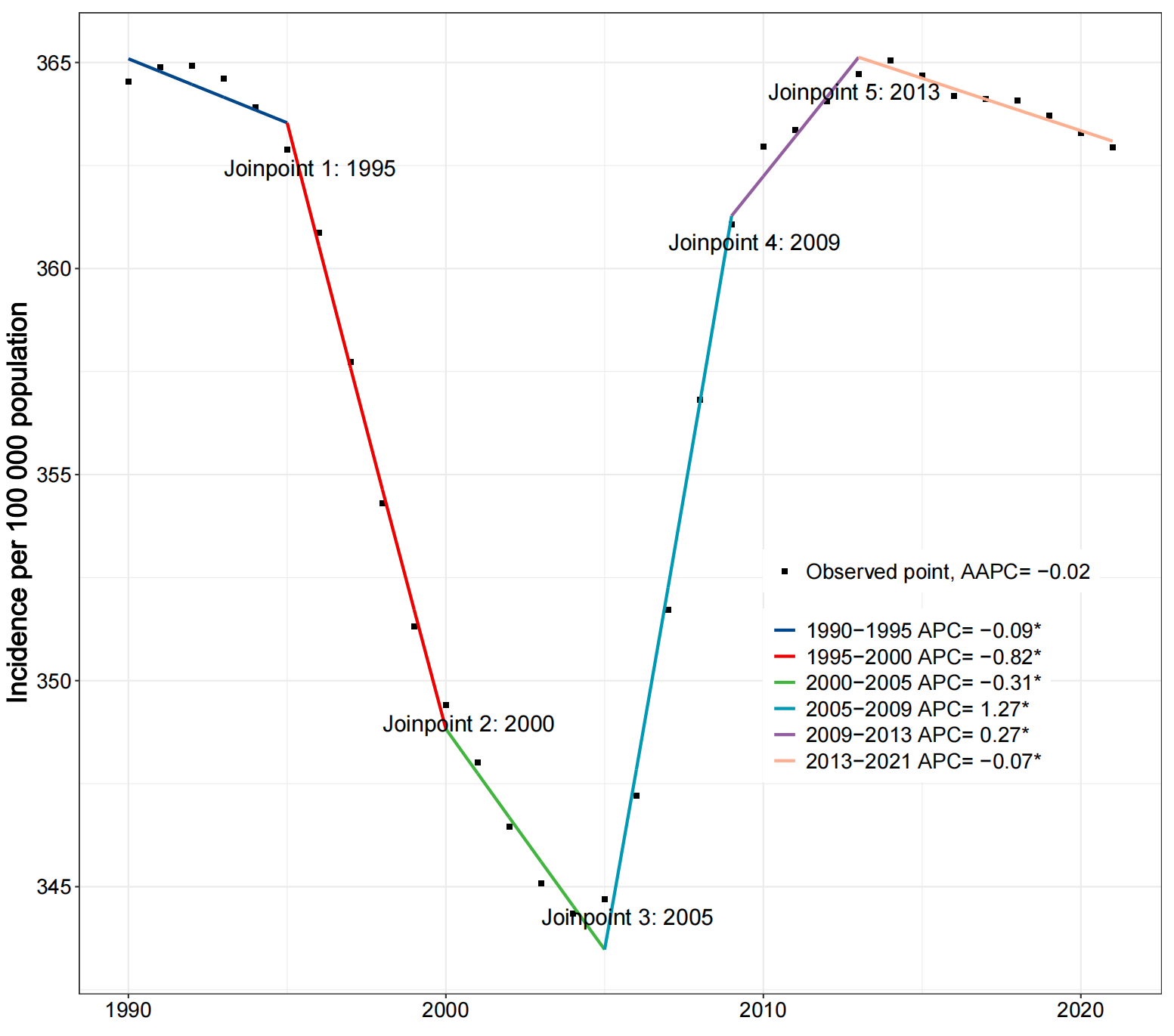


Supplement Figure 5. Joinpoint regression analysis of global neck pain prevalence in female in adolescents and young adults aged 10-24 years from 1990 to 2021.
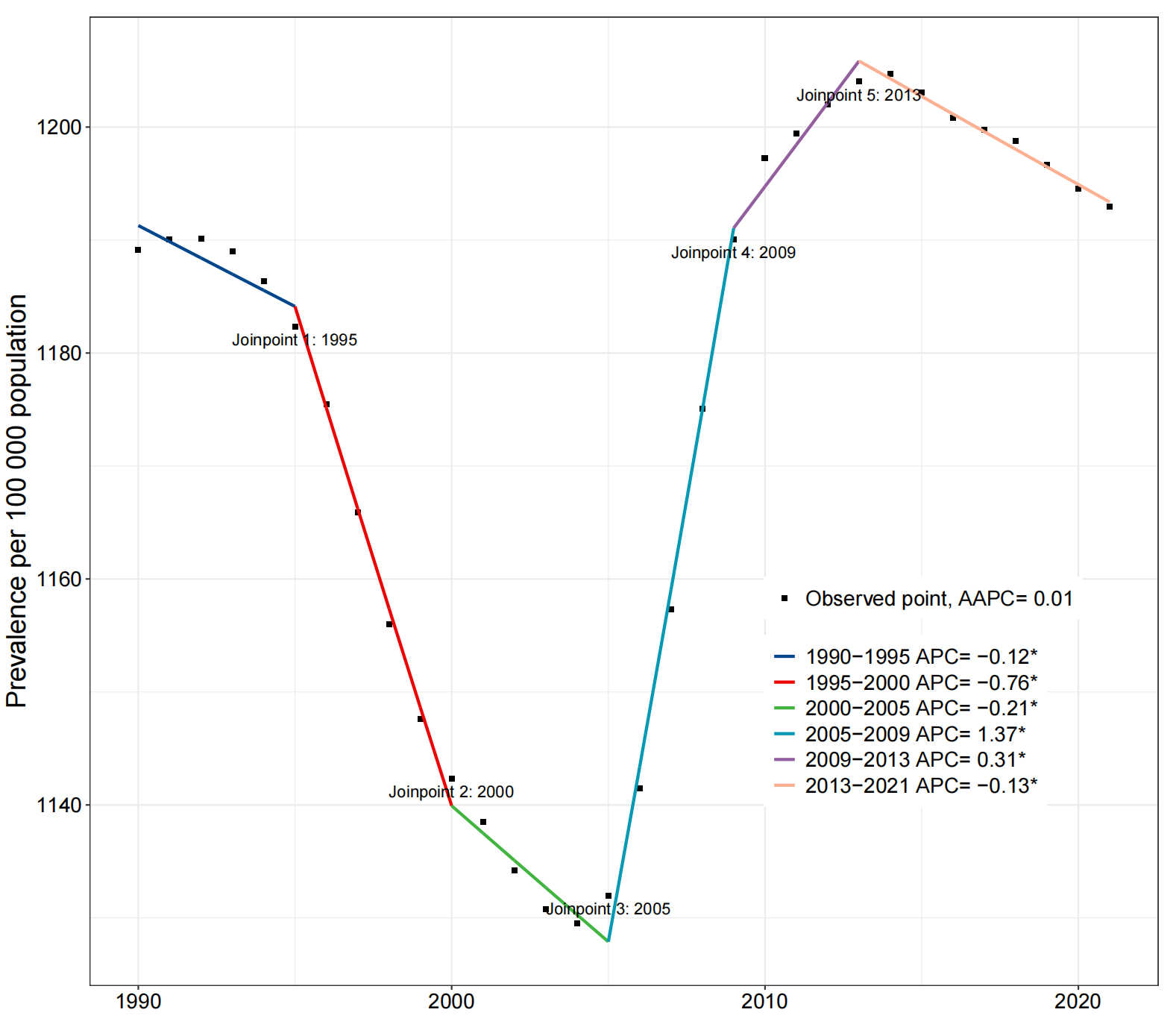


Supplement Figure 6. Joinpoint regression analysis of global neck pain YLDs in female in adolescents and young adults aged 10-24 years from 1990 to 2021.


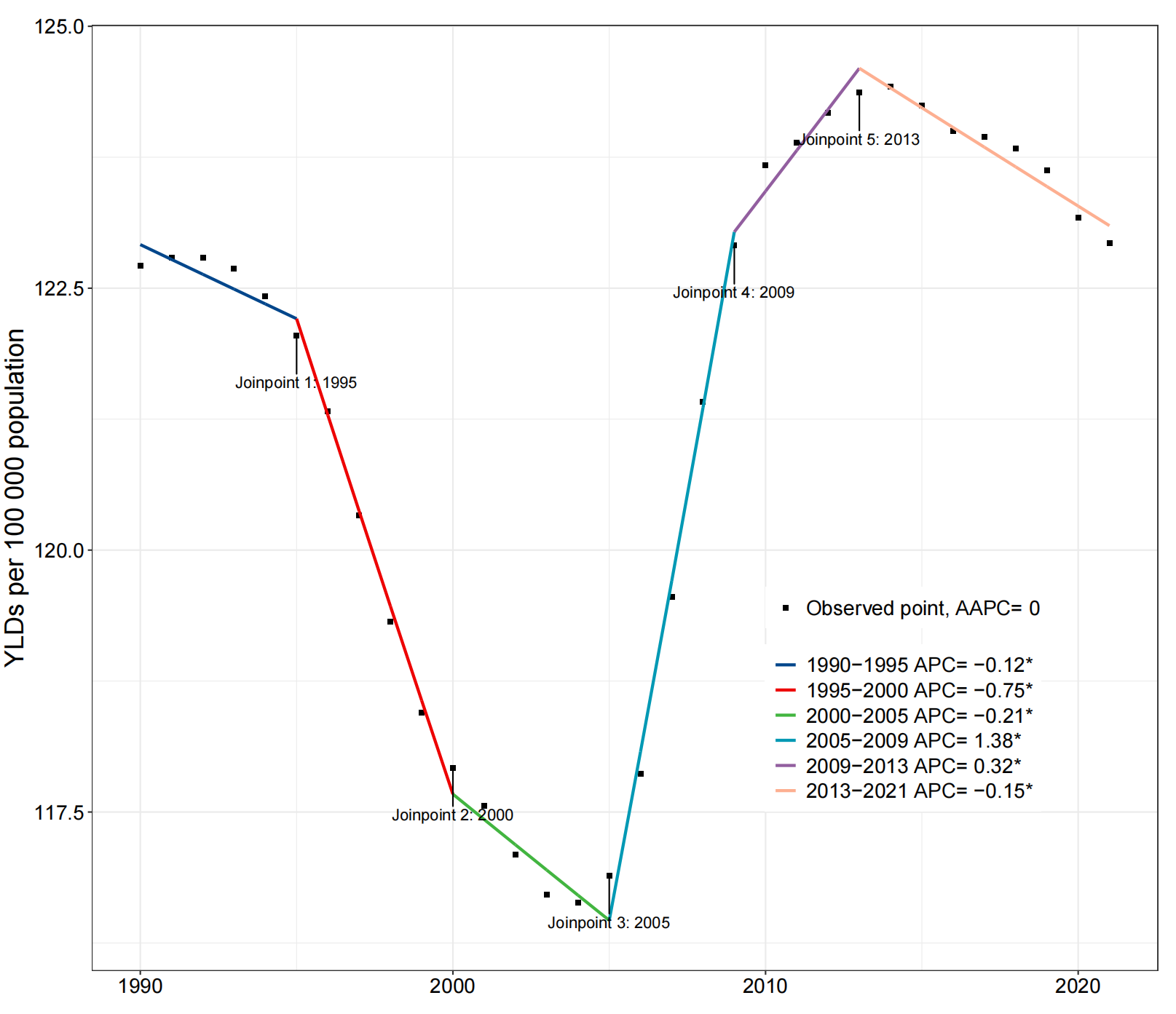


Supplement Figure 7. Global map of 2021 incidence of neck pain in male (per 100,000 population) in adolescents and young adults aged 10-24 years from 1990 to 2021.


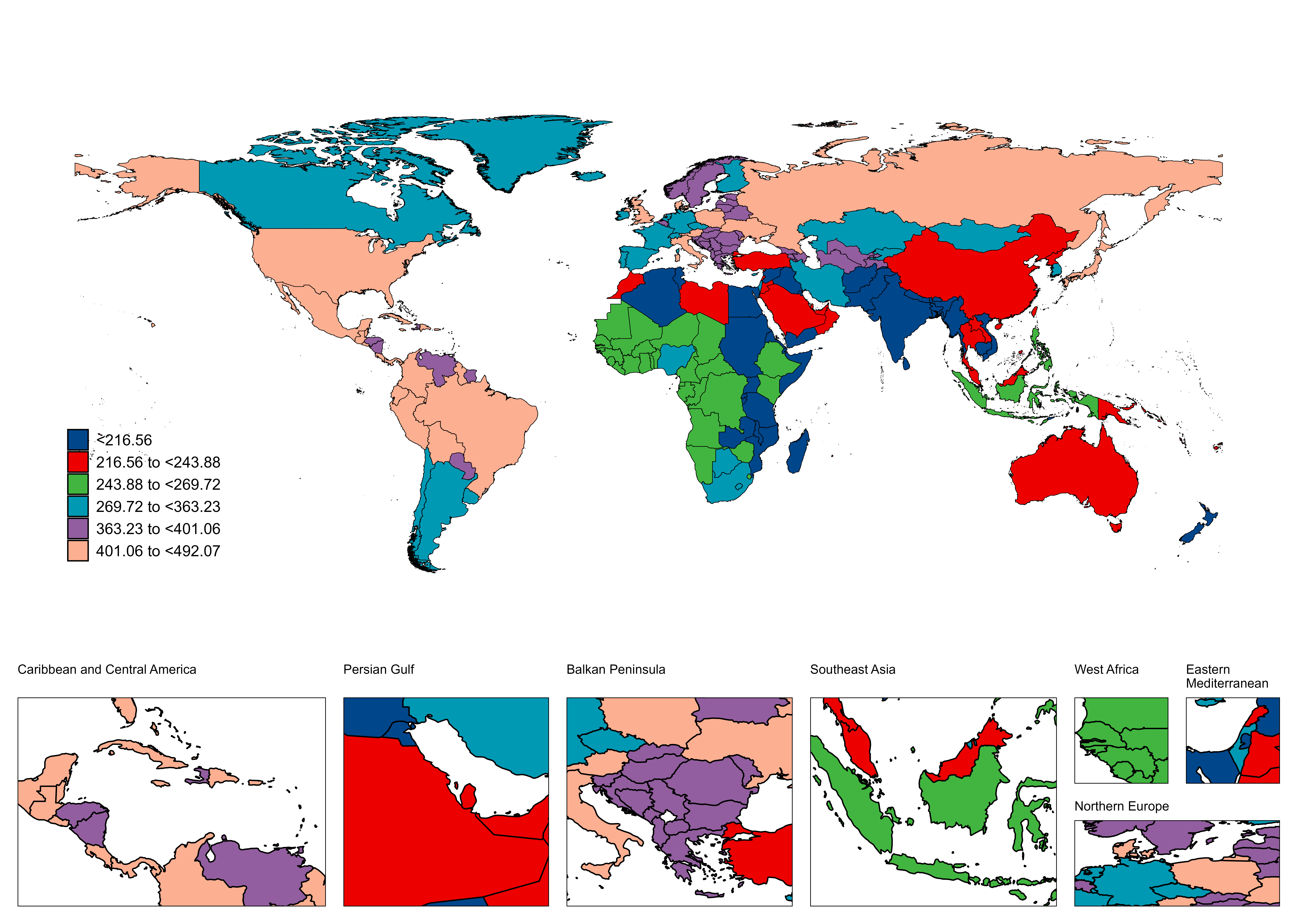


Supplement Figure 8. Global map of 2021 prevalence of neck pain in male (per 100,000 population) in adolescents and young adults aged 10-24 years from 1990 to 2021.


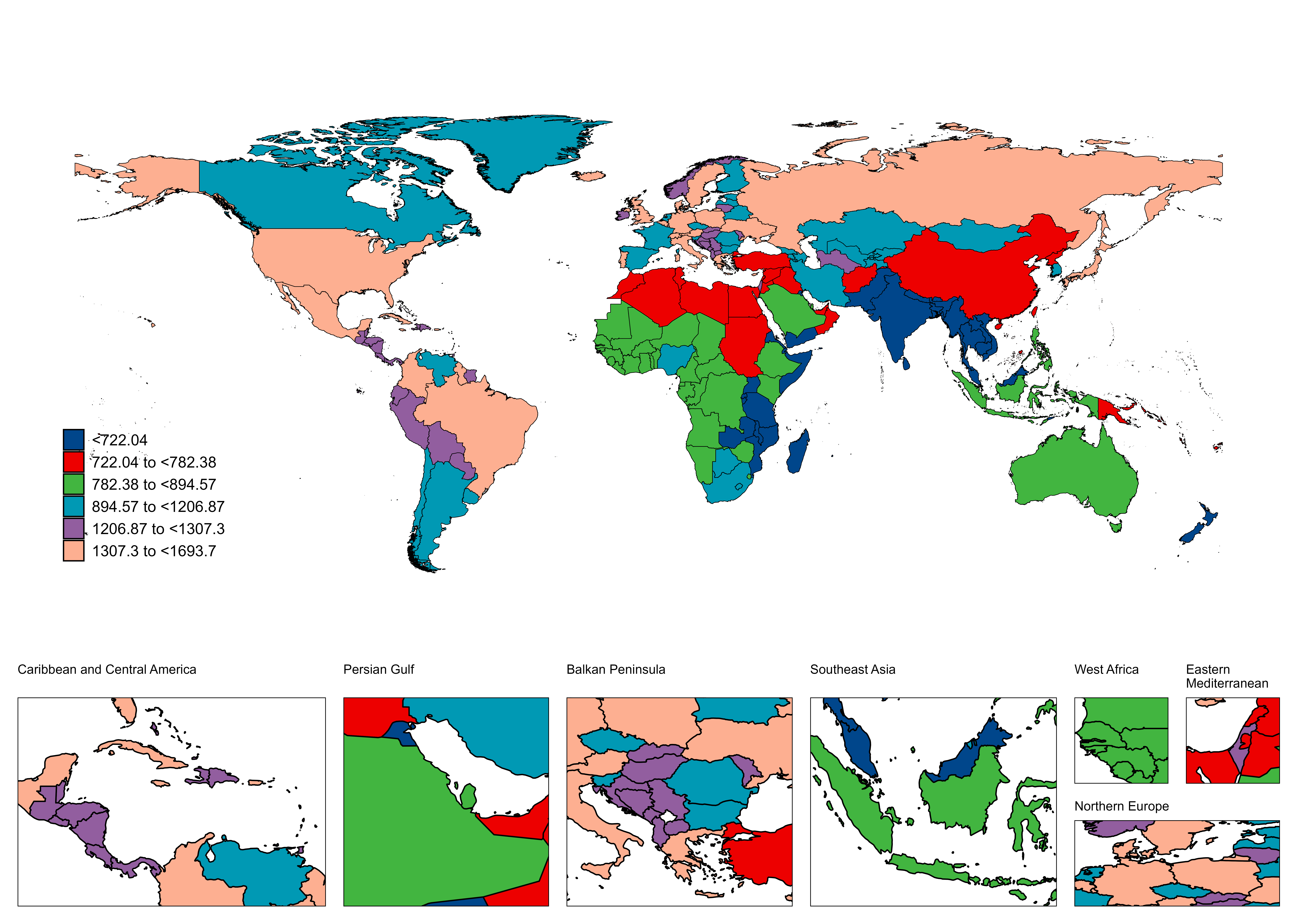


Supplement Figure 9. Global map of 2021 YLDs of neck pain in male (per 100,000 population) in adolescents and young adults aged 10-24 years from 1990 to 2021.


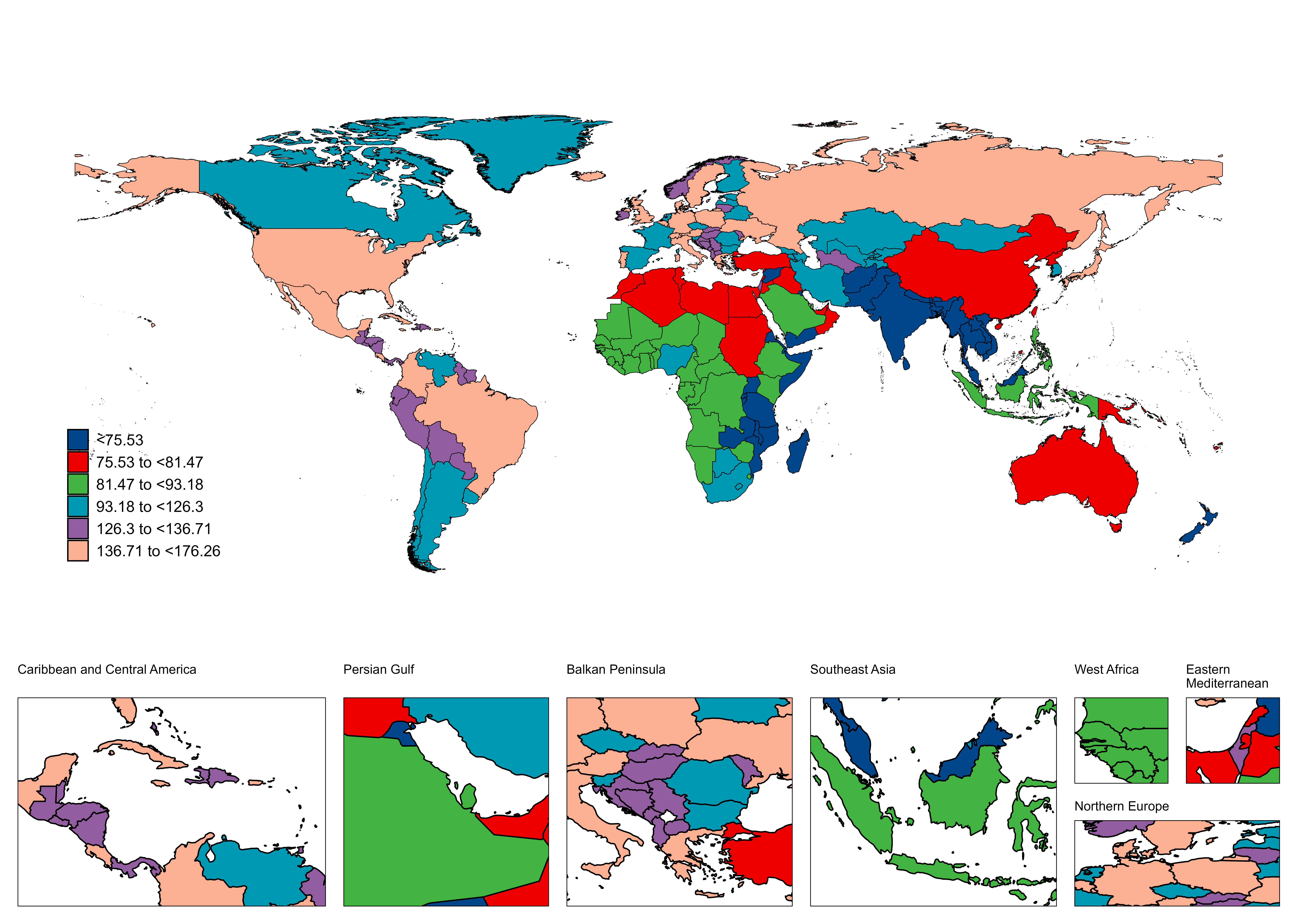


Supplement Figure 10. Global map of 2021 incidence of neck pain in female (per 100,000 population) in adolescents and young adults aged 10-24 years from 1990 to 2021.


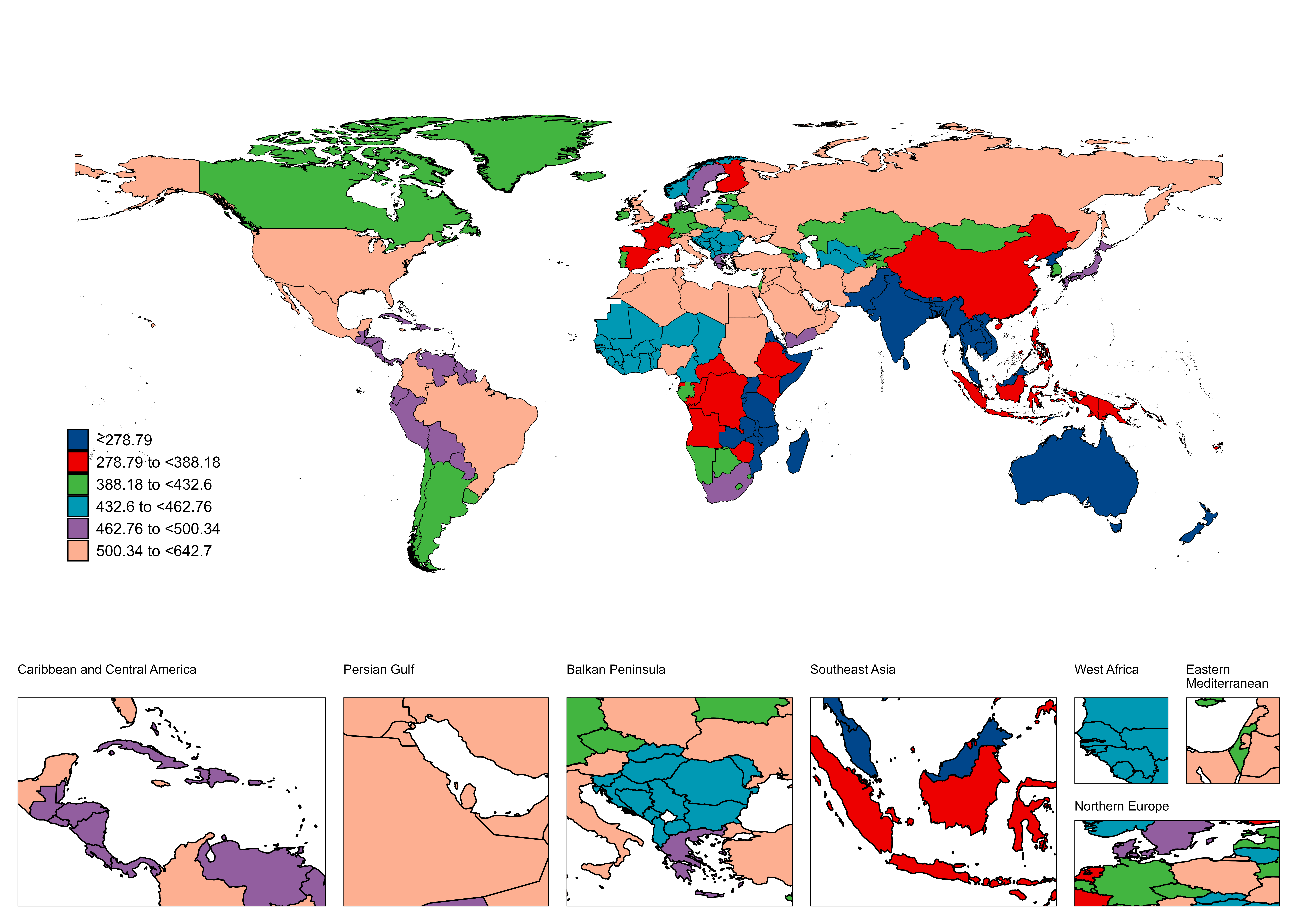


Supplement Figure 11. Global map of 2021 prevalence of neck pain in female (per 100,000 population) in adolescents and young adults aged 10-24 years from 1990 to 2021.


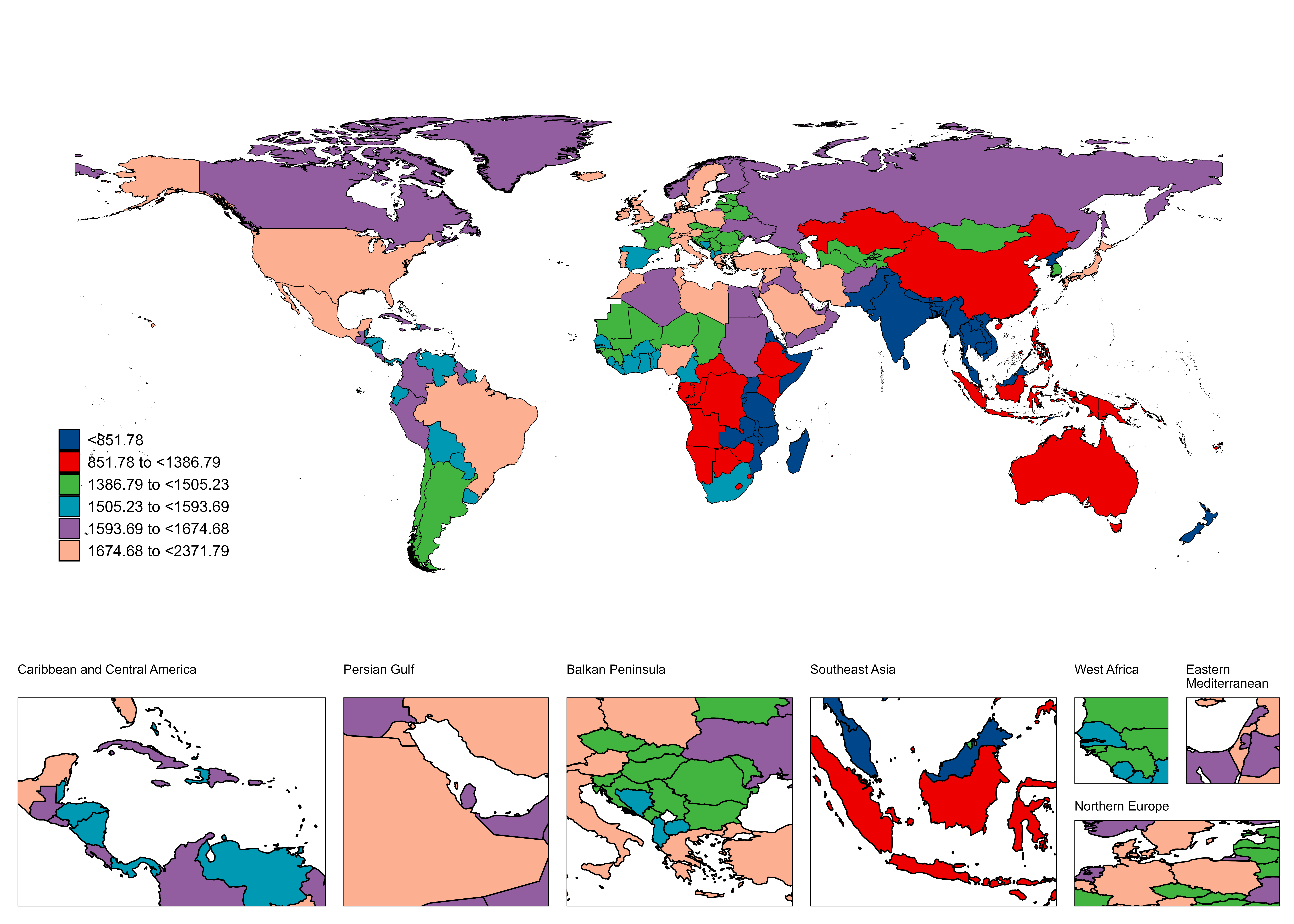


Supplement Figure 12. Global map of 2021 prevalence of neck pain in female (per 100,000 population) in adolescents and young adults aged 10-24 years from 1990 to 2021.


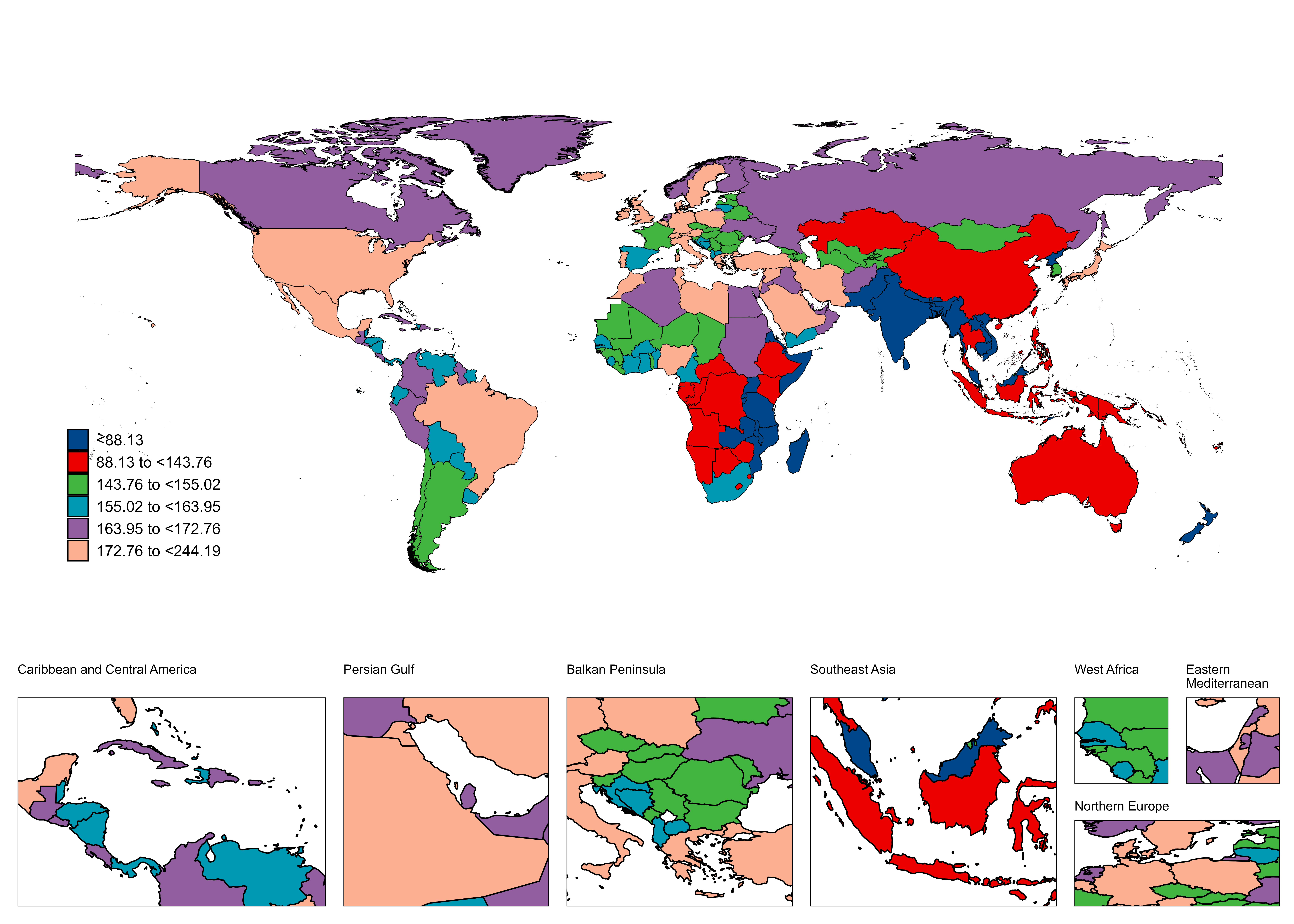


Supplement Figure 13. Joinpoint regression analysis of global neck pain incidence in adolescents and young adults aged 10-14 years from 1990 to 2021.


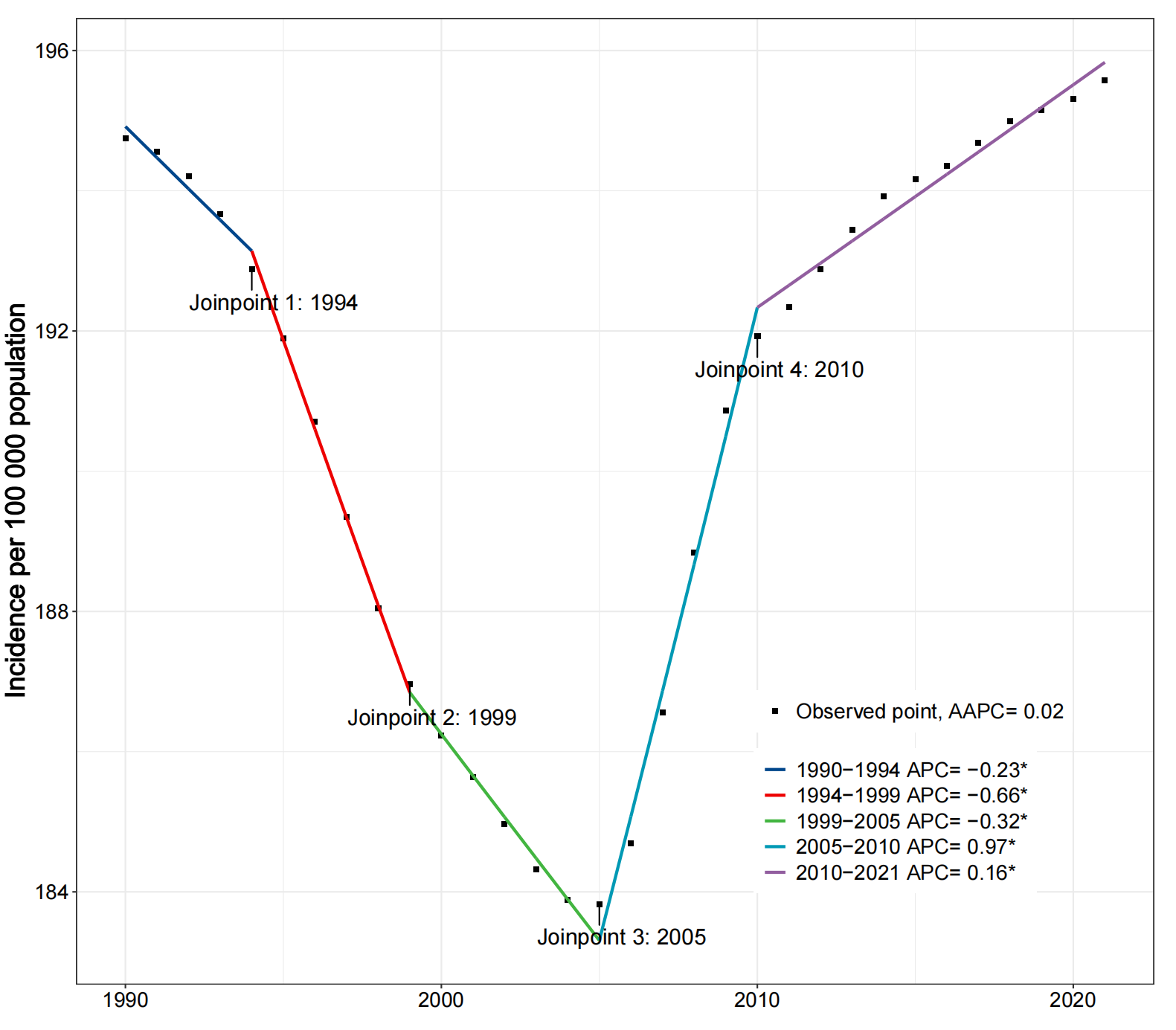


Supplement Figure 14. Joinpoint regression analysis of global neck pain incidence in adolescents and young adults aged 15-19 years from 1990 to 2021.


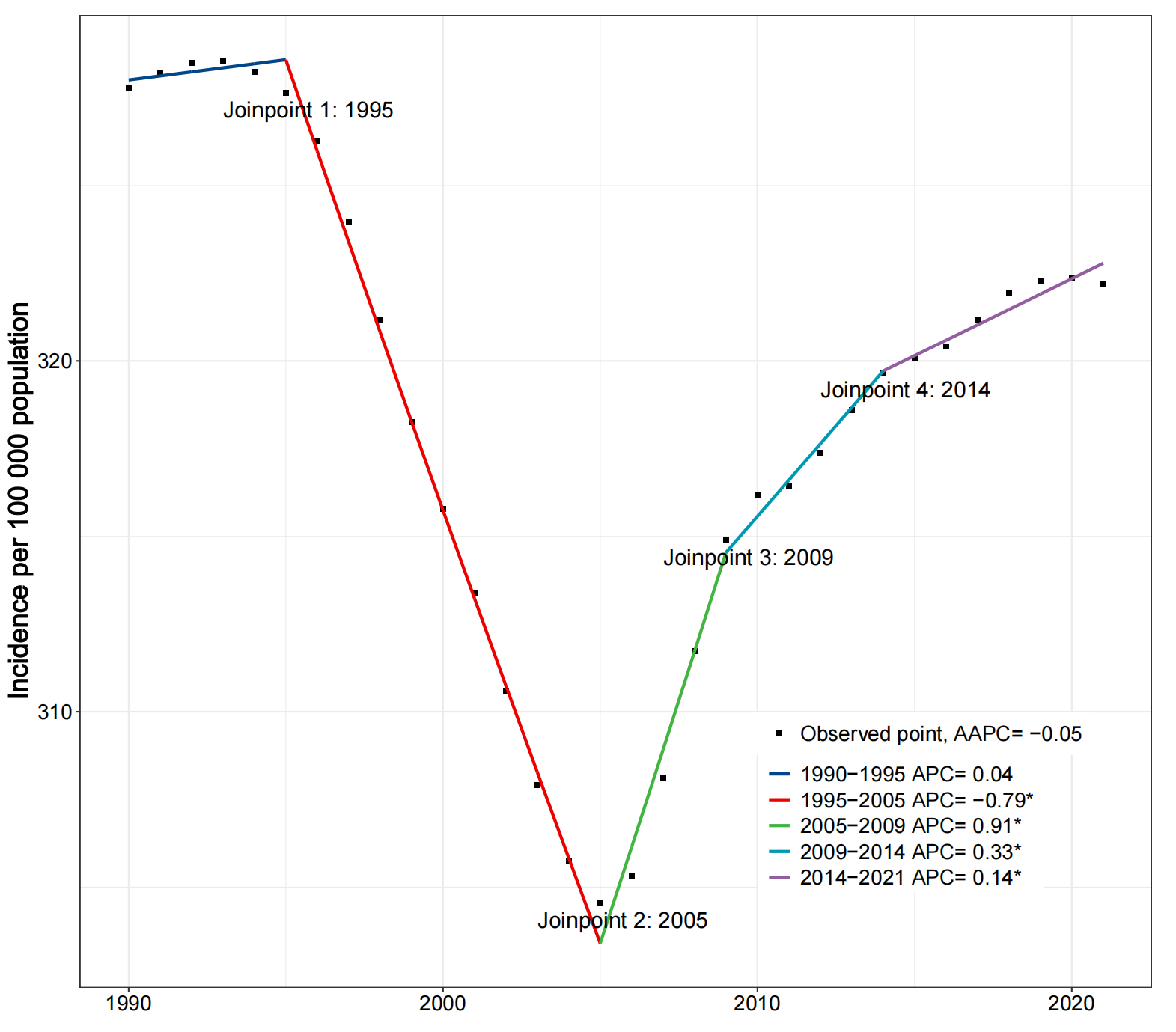


Supplement Figure 15. Joinpoint regression analysis of global neck pain incidence in adolescents and young adults aged 20-24 years from 1990 to 2021.


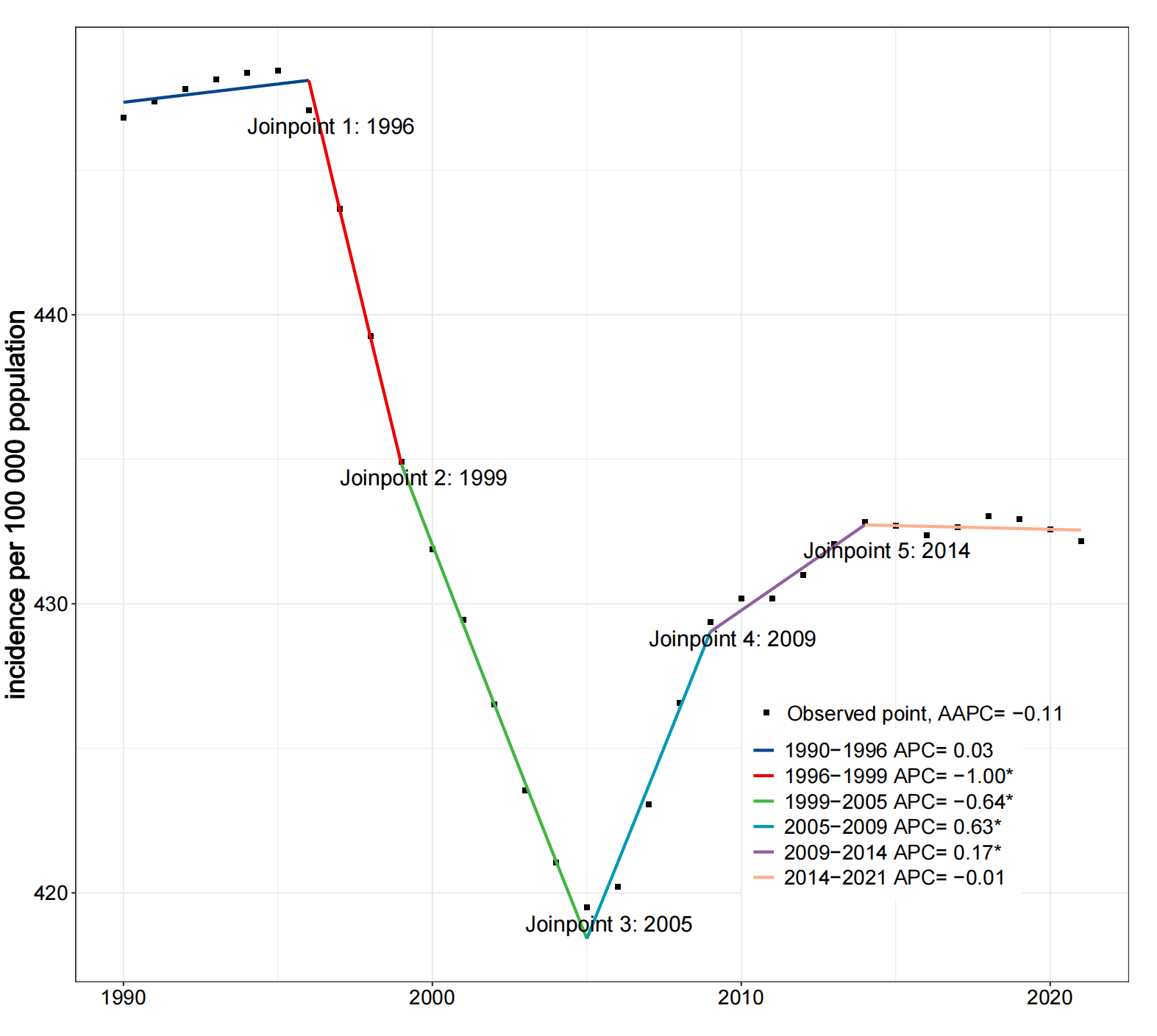


Supplement Figure 16. Joinpoint regression analysis of global neck pain prevalence in adolescents and young adults aged 10-14 years from 1990 to 2021.


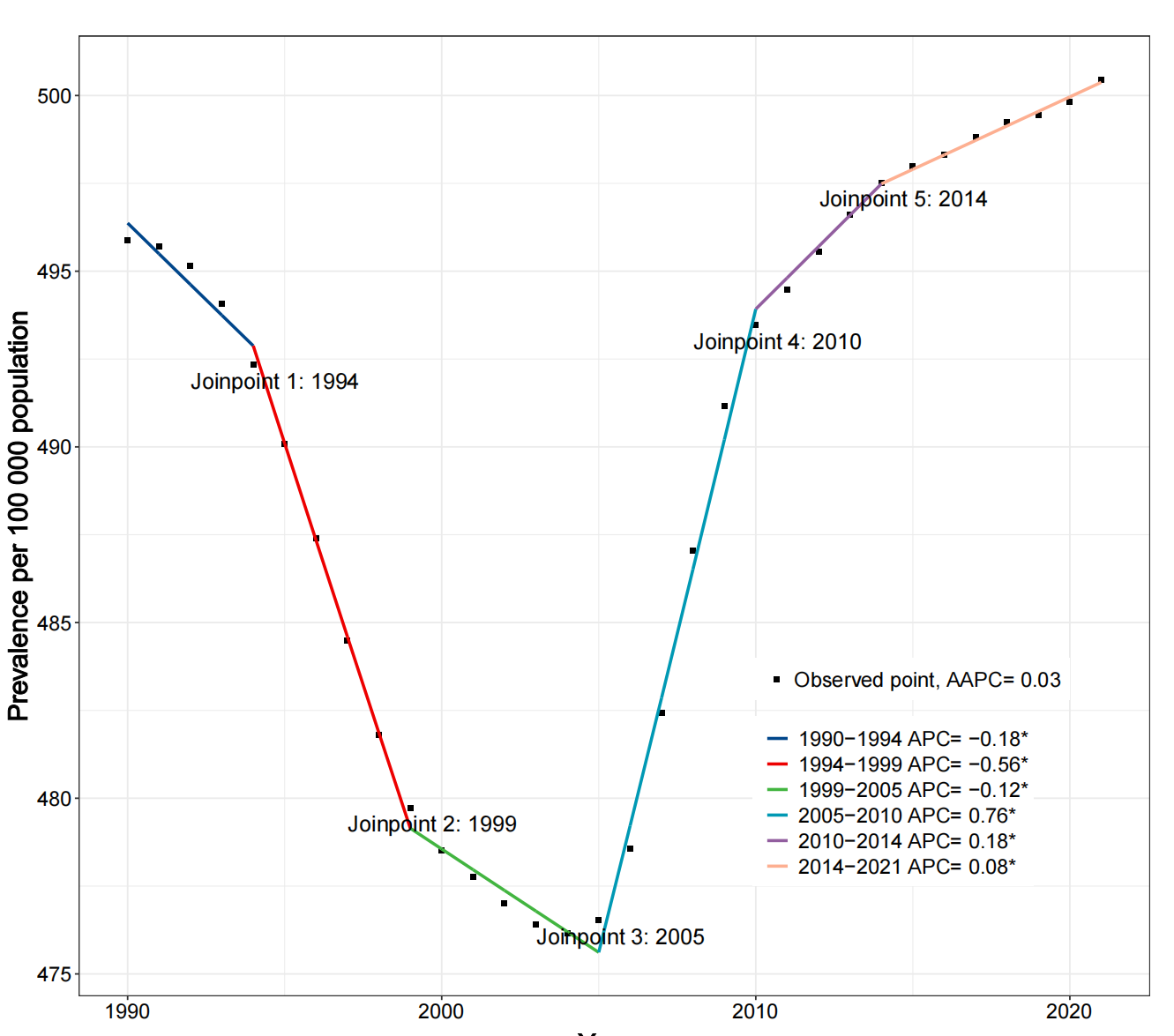


Supplement Figure 17. Joinpoint regression analysis of global neck pain prevalence in adolescents and young adults aged 15-19 years from 1990 to 2021.


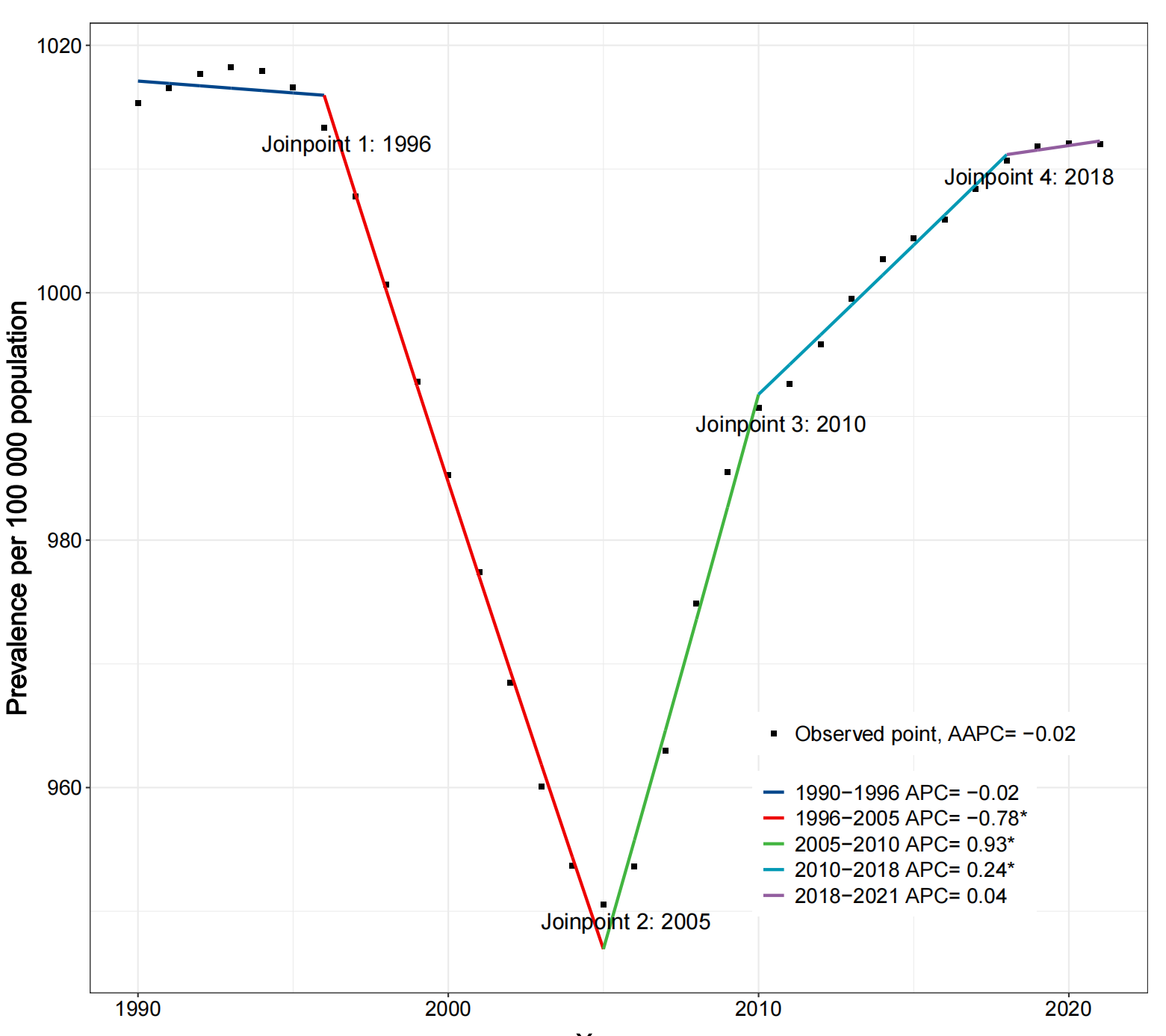


Supplement Figure 18. Joinpoint regression analysis of global neck pain prevalence in adolescents and young adults aged 20-24 years from 1990 to 2021.


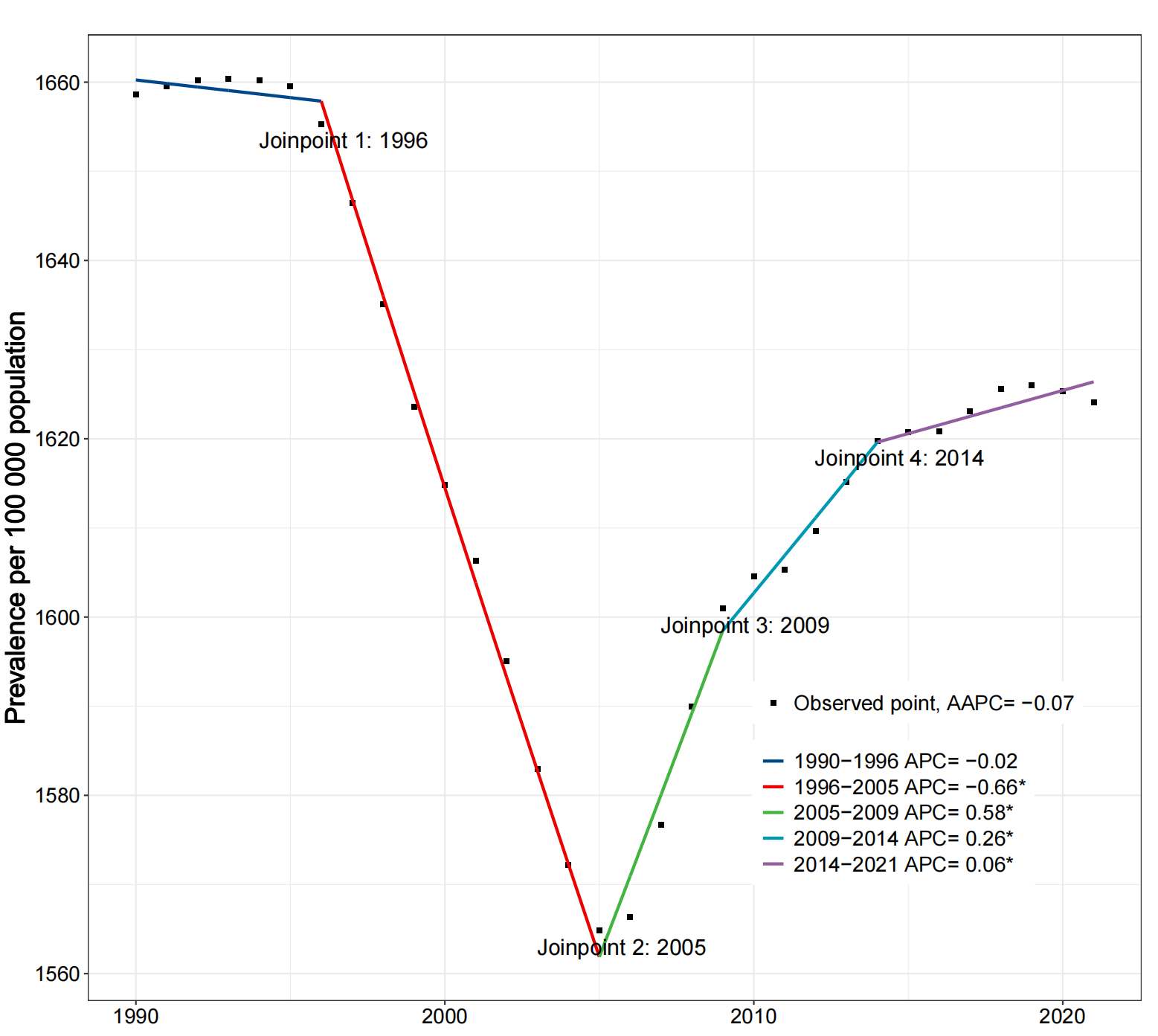


Supplement Figure 19. Joinpoint regression analysis of global neck pain YLDs in adolescents and young adults aged 10-14 years from 1990 to 2021.


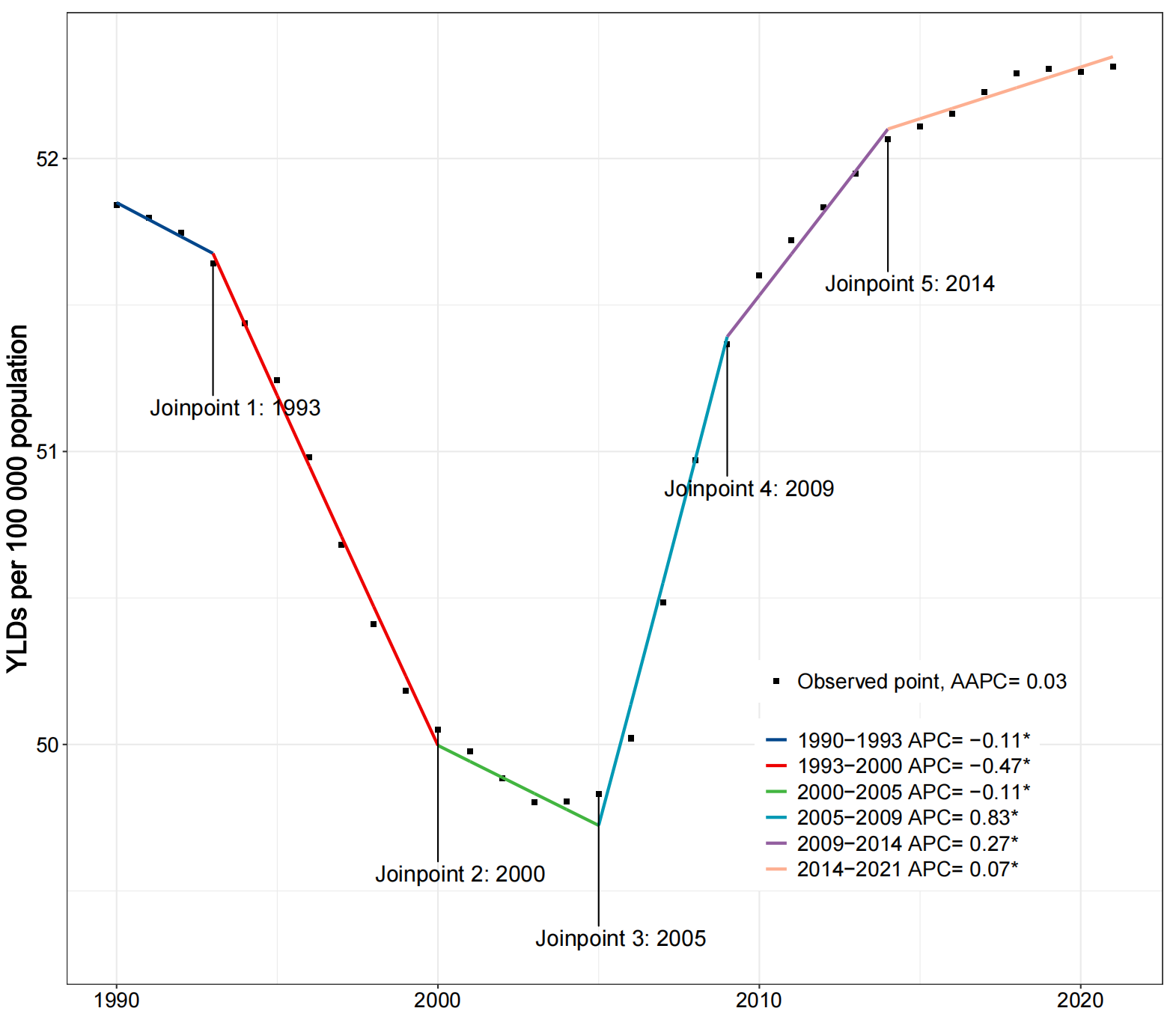


Supplement Figure 20. Joinpoint regression analysis of global neck pain YLDs in adolescents and young adults aged 15-19 years from 1990 to 2021.


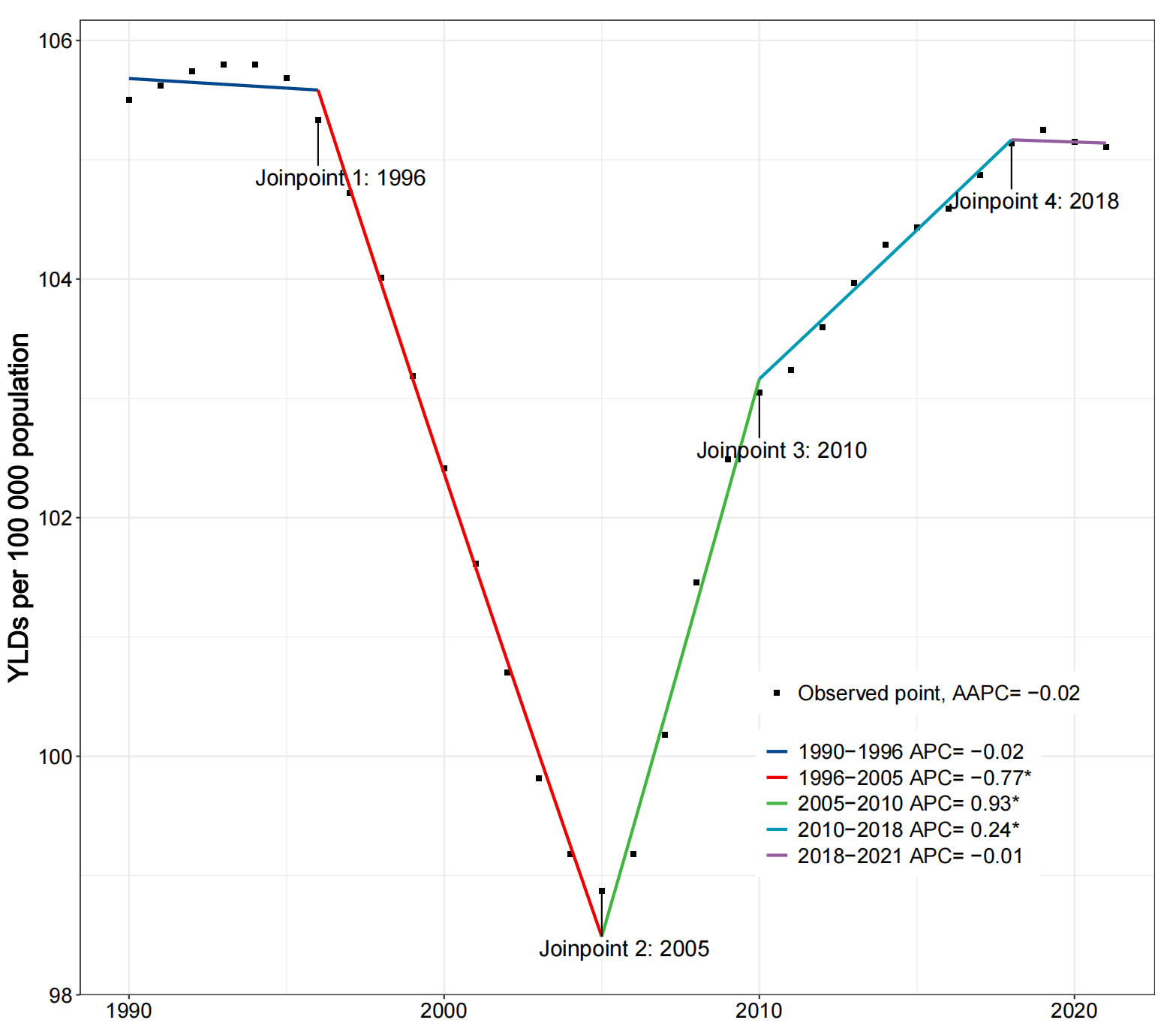


Supplement Figure 21. Joinpoint regression analysis of global neck pain YLDs in adolescents and young adults aged 20-24 years from 1990 to 2021.


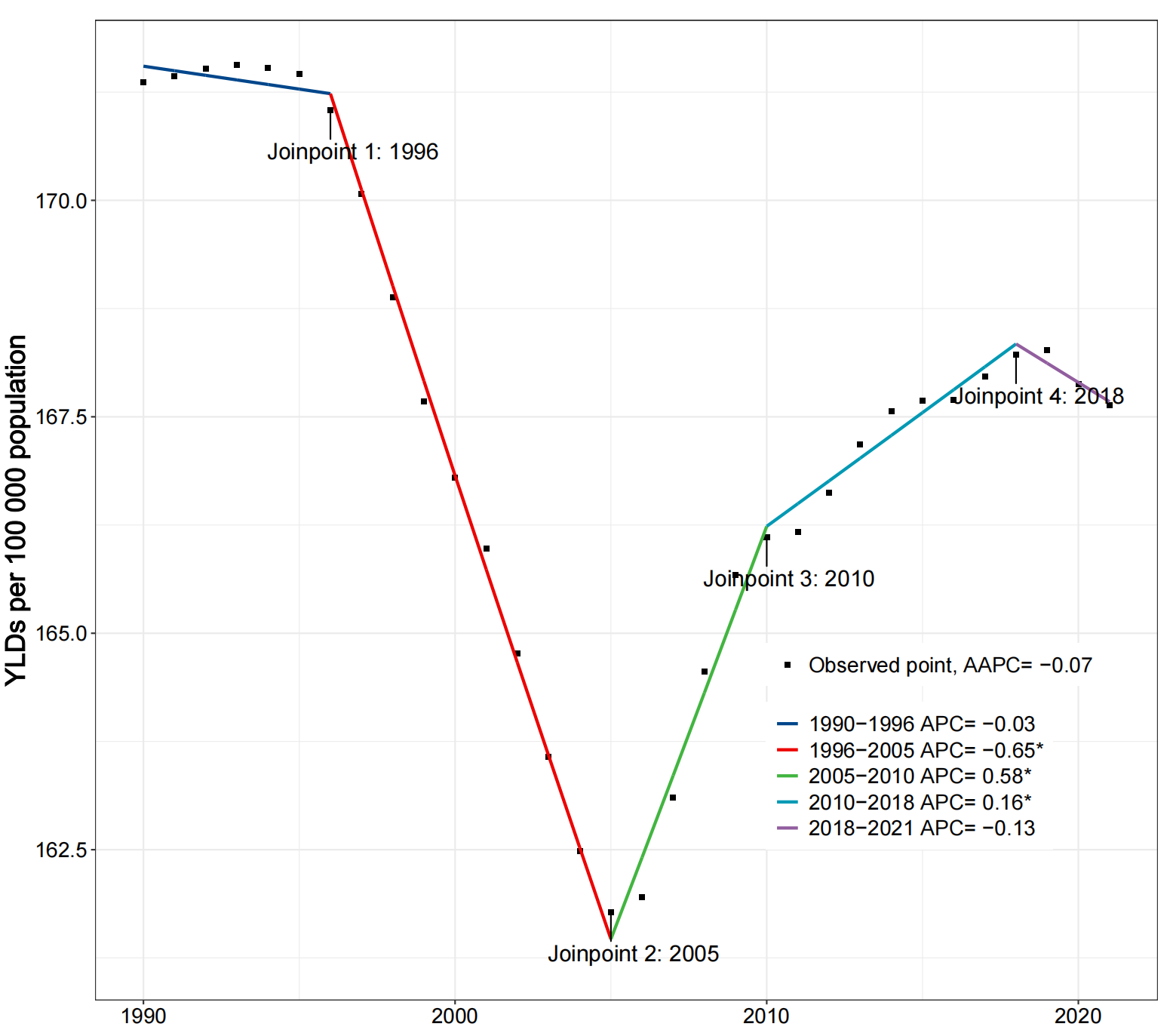


Supplement Figure 22. Global map of 2021 incidence of global neck pain incidence (per 100,000 population) in adolescents and young adults aged 10-14 years.


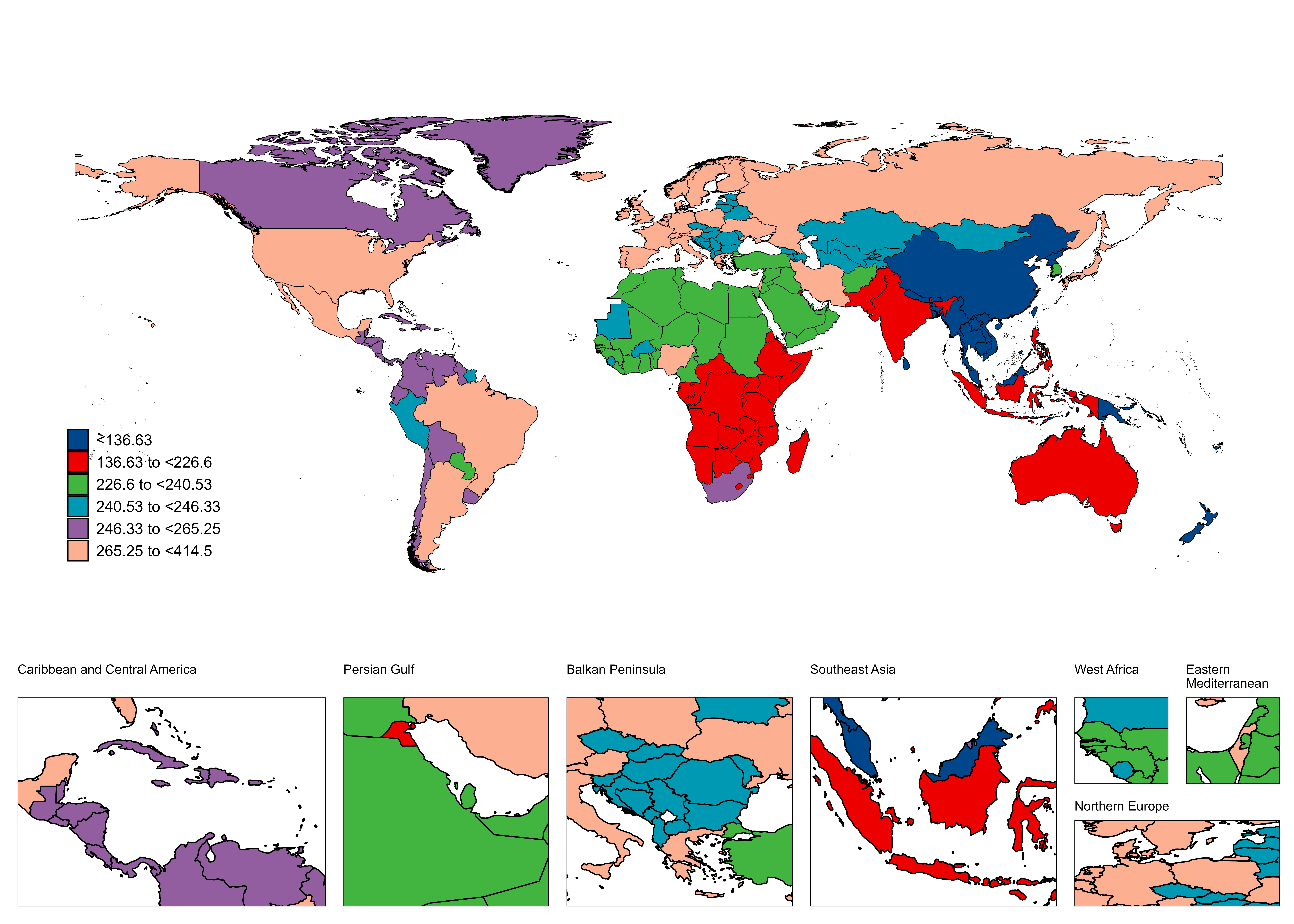


Supplement Figure 23. Global map of 2021 incidence of global neck pain incidence (per 100,000 population) in adolescents and young adults aged 15-19 years.


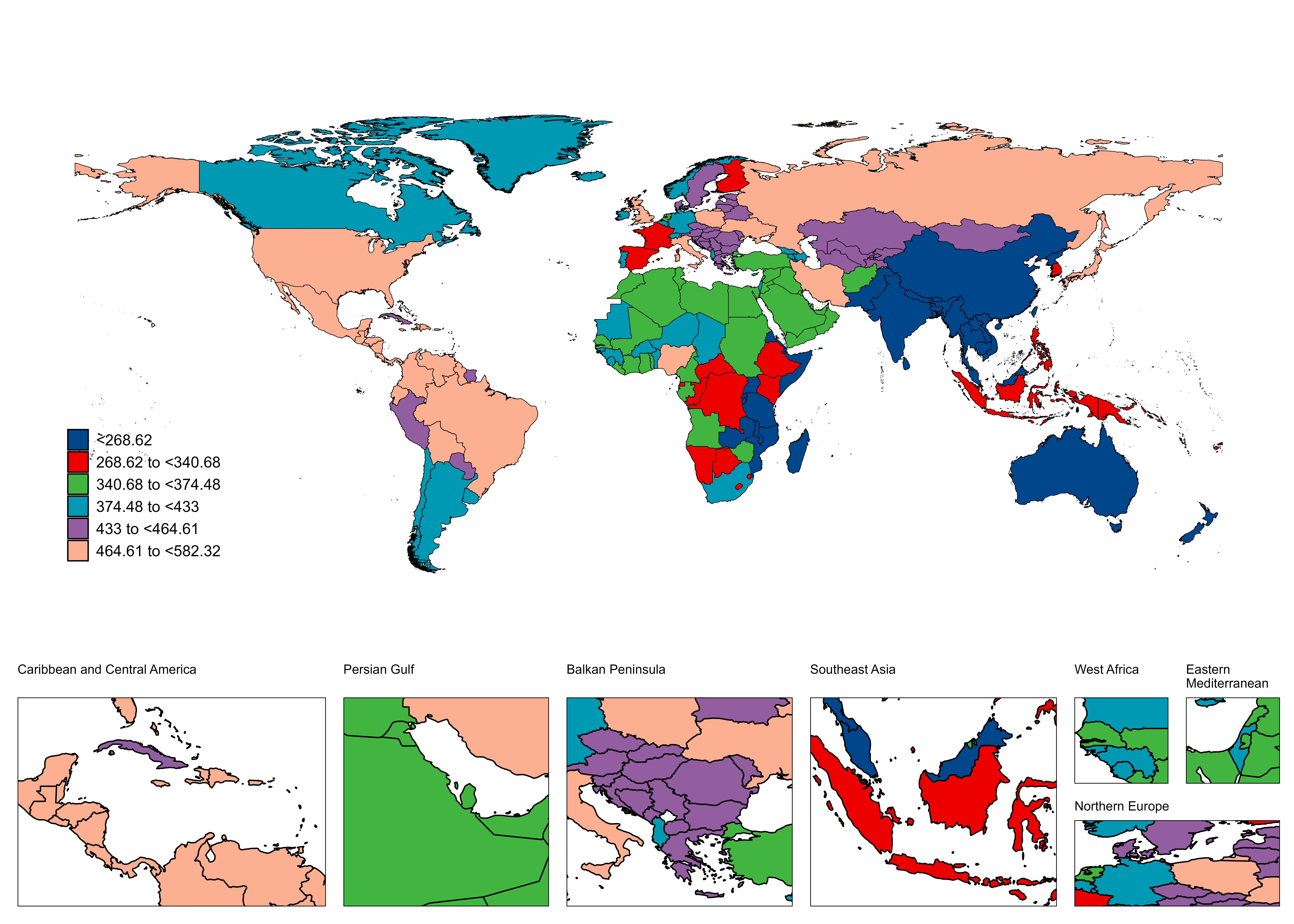


Supplement Figure 24. Global map of 2021 incidence of global neck pain incidence (per 100,000 population) in adolescents and young adults aged 20-24 years.


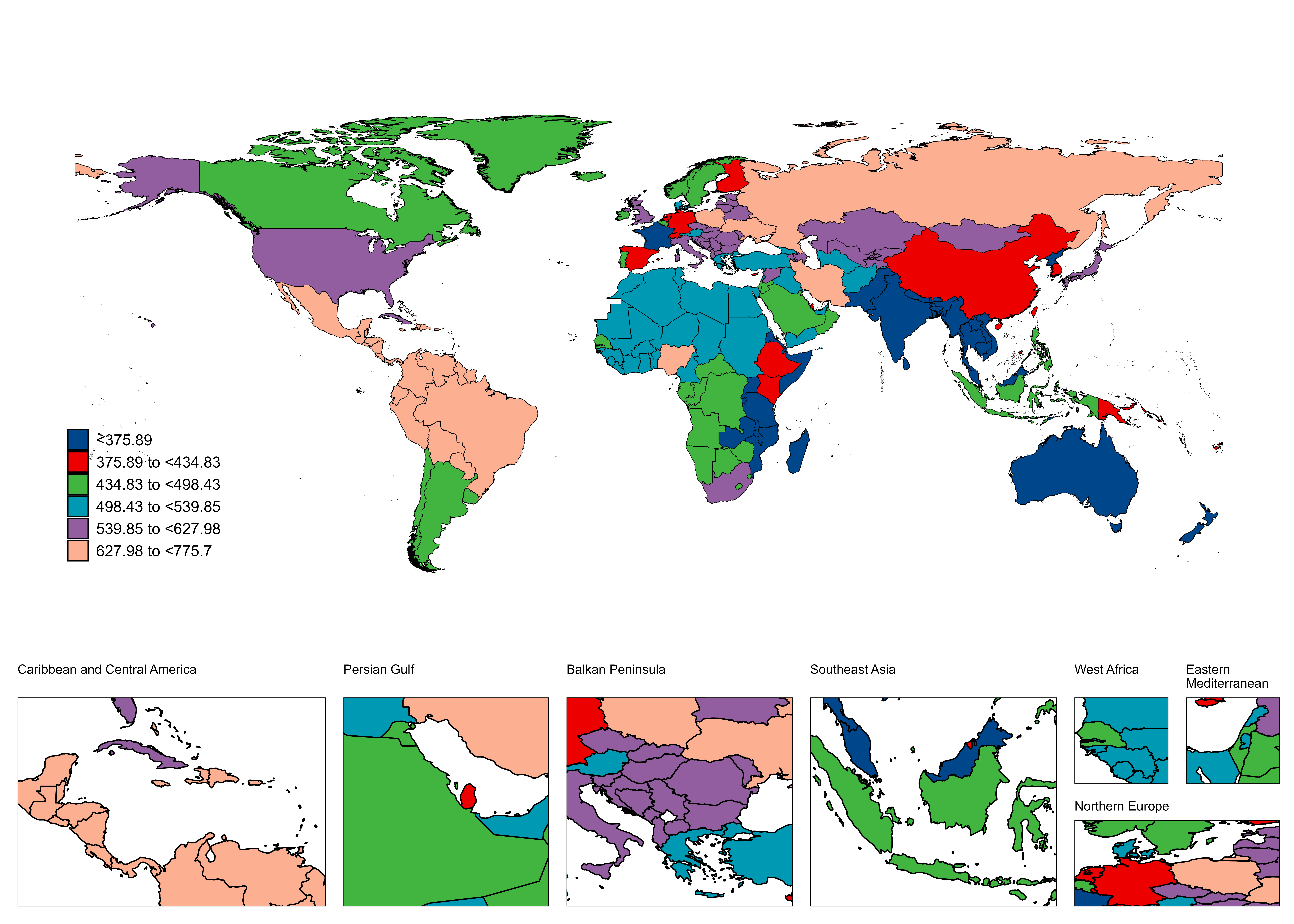


Supplement Figure 25. Global map of 2021 incidence of global neck pain prevalence (per 100,000 population) in adolescents and young adults aged 10-14 years.


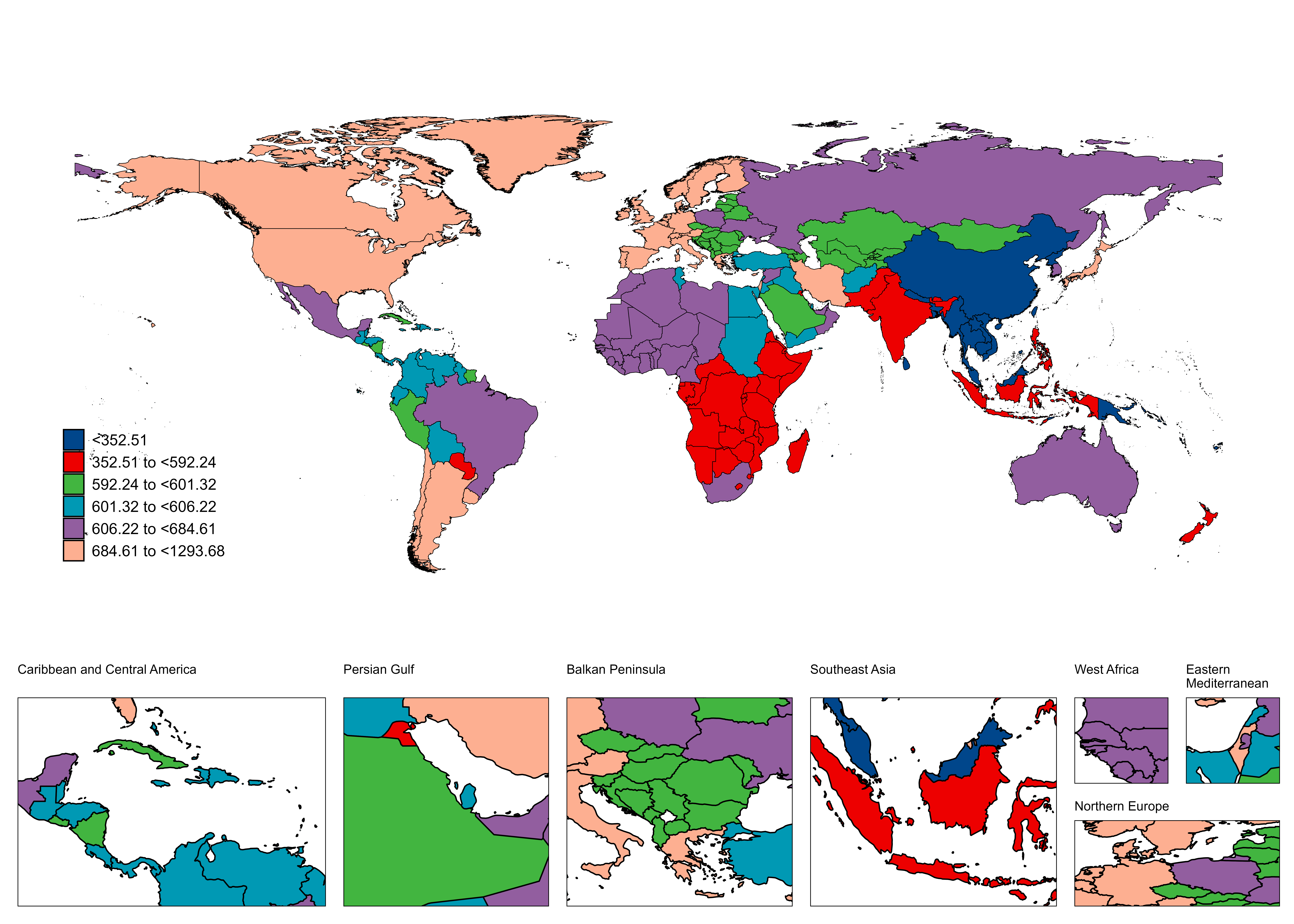


Supplement Figure 26. Global map of 2021 incidence of global neck pain prevalence (per 100,000 population) in adolescents and young adults aged 15-19 years.


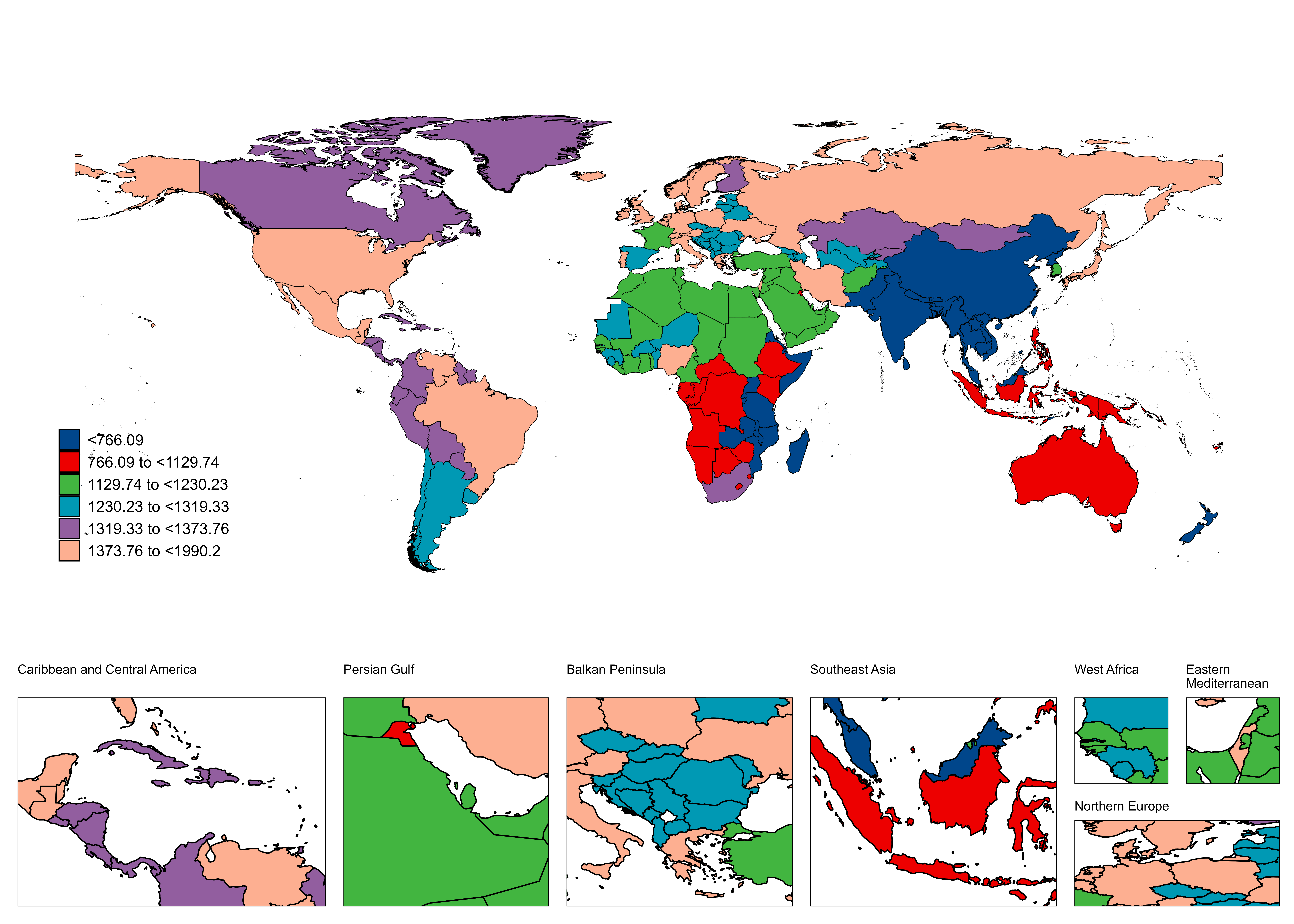


Supplement Figure 27. Global map of 2021 incidence of global neck pain prevalence (per 100,000 population) in adolescents and young adults aged 20-24 years.


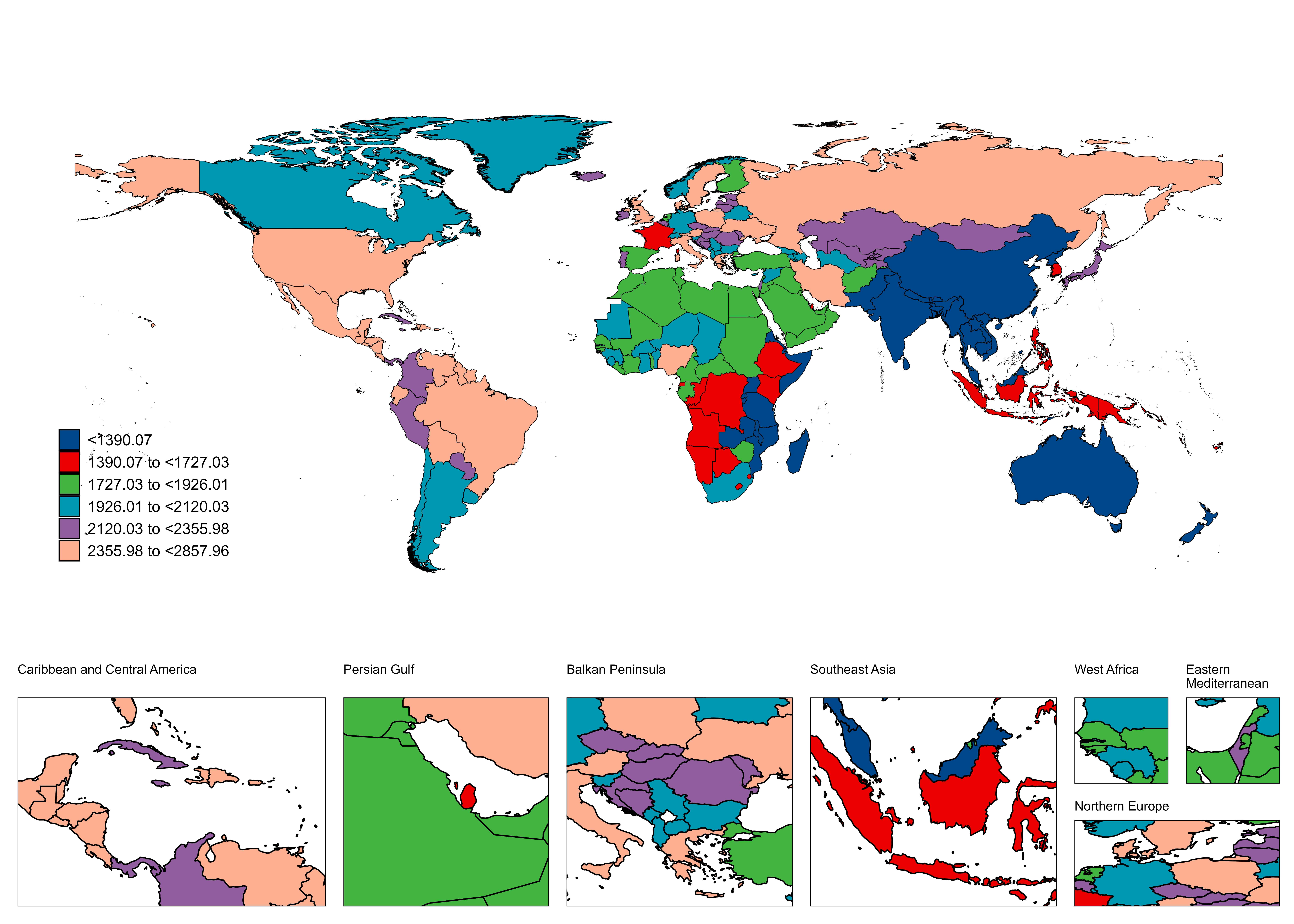


Supplement Figure 28. Global map of 2021 incidence of global neck pain YLDs (per 100,000 population) in adolescents and young adults aged 10-14 years.


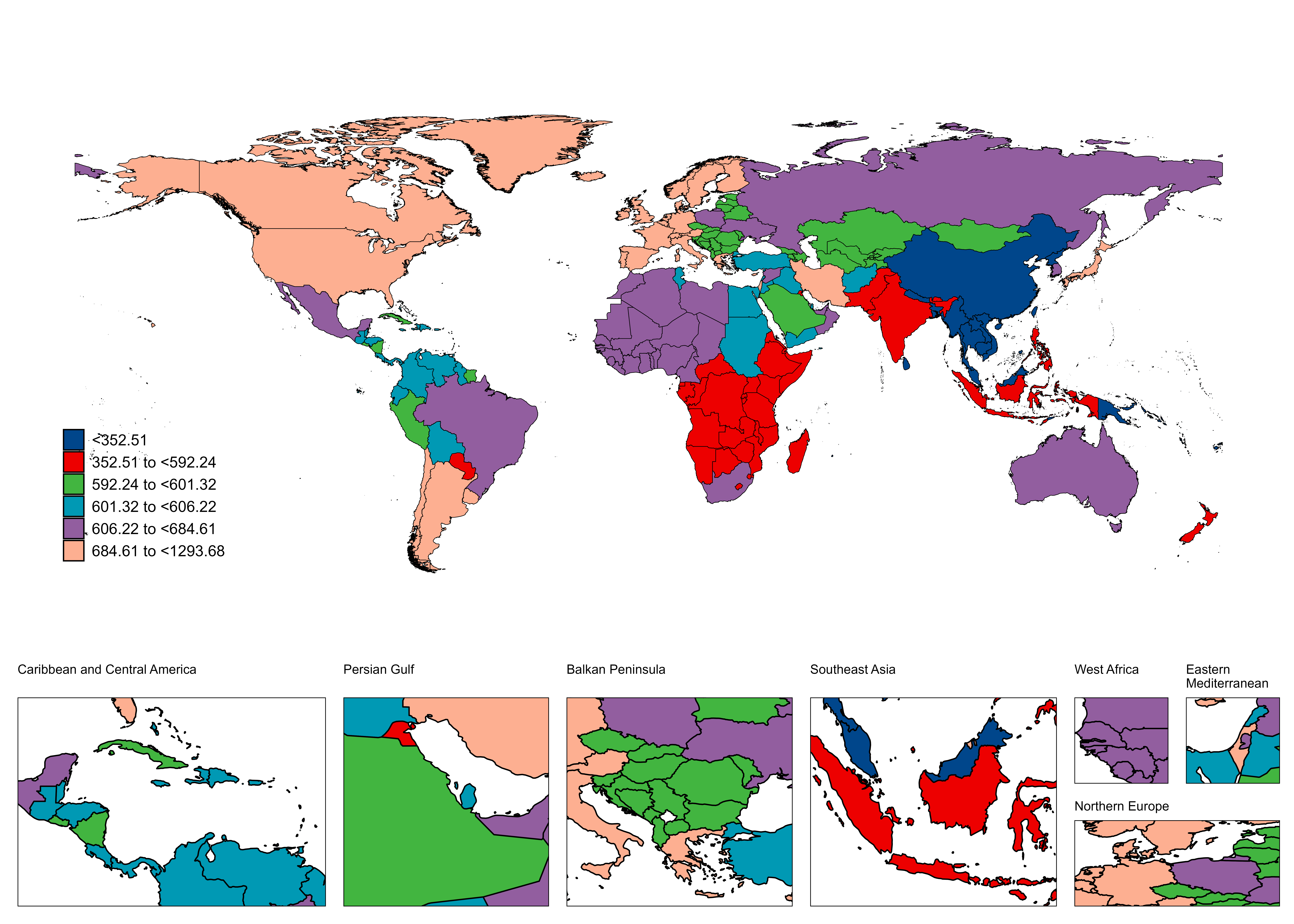


Supplement Figure 29. Global map of 2021 incidence of global neck pain YLDs (per 100,000 population) in adolescents and young adults aged 15-19 years.


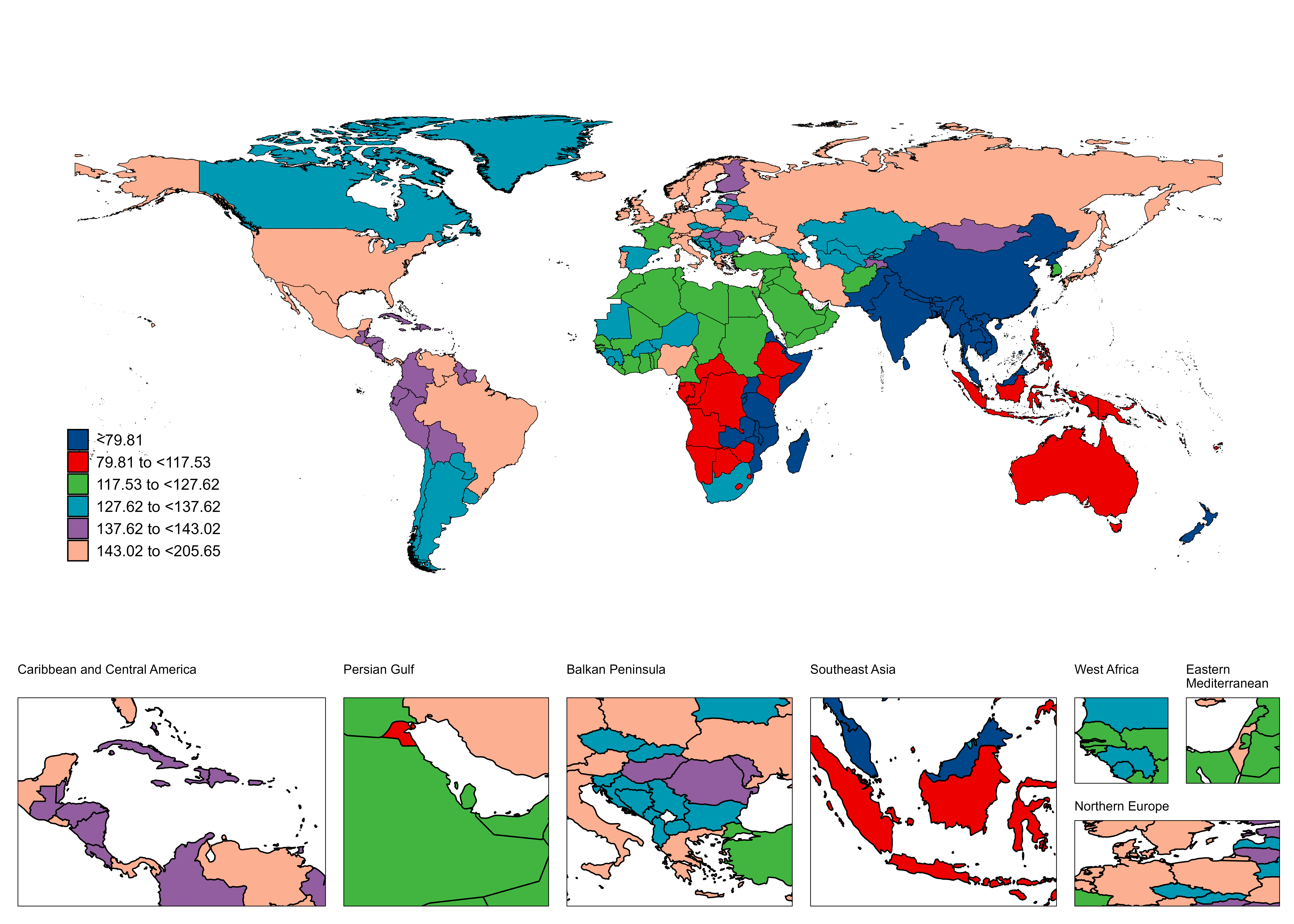


Supplement Figure 30. Global map of 2021 incidence of global neck pain YLDs (per 100,000 population) in adolescents and young adults aged 20-24 years.


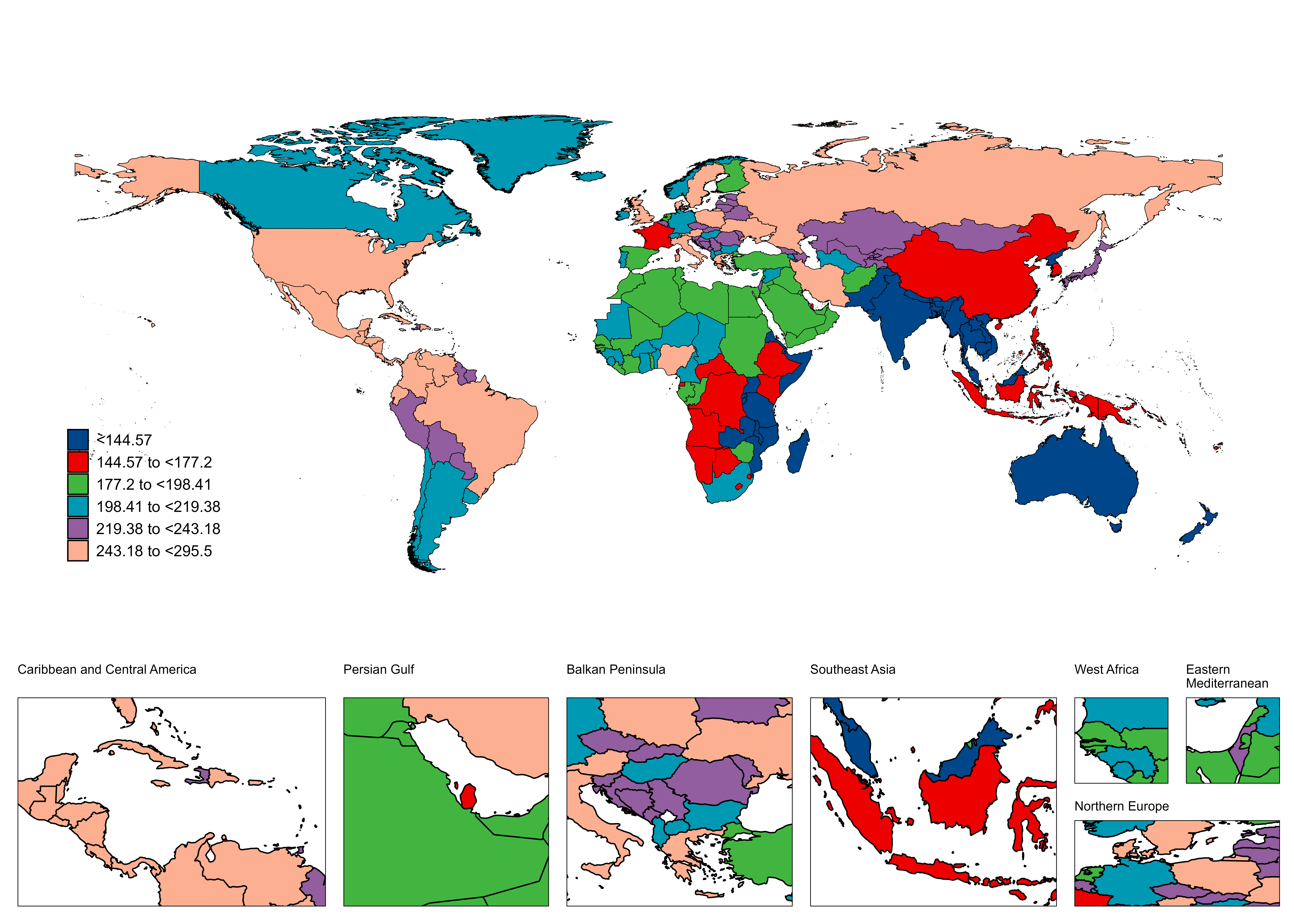


Supplementary Table 1: The Incidence of neck pain and their AAPCs from 1990 to 2021 at the global and regional levels.

|  |  | **Incidence** |  |  |  |  |  |  |
| --- | --- | --- | --- | --- | --- | --- | --- | --- |
|  |  | Cases (n), 1990 | Age standardised rate in 1990 (per 100,000) | Cases (n), 2021 | Age standardised rate in 2021 (per 100,000) | AAPC, 1990–2021 | p value |  |
|  | Global | 4944467  (2843903 to 7716711) | 317  (138 to 575) | 5894956  (3429037 to 9131211) | 311  (137 to 561) | -0.08  (-0.11 to -0.05) | <0.001 |  |
|  | Sex |  |  |  |  |  |  |  |
|  | Male | 2169939 (1247208 to 3346791) | 275  (120 to 487) | 2554753 (1493485 to 3921241) | 263  (116 to 495) | -0.15  (-0.17 to -0.12) | <0.001 |  |
|  | Female | 2774528 (1595002 to 4331081) | 361  (157 to 487) | 3340202  (1944353 to 5194835) | 361  (158 to 495) | -0.02  (-0.04 to 0) | 0.125 |  |
|  | Age group, years | | | | | | |  |
|  | 10-14 | 1043216 (517905 to 1933902) | 195  (97 to 487) | 1303744  (649522 to 2410679) | 196  (97 to 495) | 0.02  (-0.01 to 0.04) | 0.203 |  |
|  | 15-19 | 1702521 (793059 to 3243068) | 328  (153 to 487) | 2010492  (932658 to 3781891) | 322  (149 to 495) | -0.05  (-0.08 to -0.03) | <0.001 |  |
|  | 20-24 | 2198731  (833659 to 3778872) | 447  (169 to 487) | 2580720  (1002594 to 4431808) | 432  (168 to 495) | -0.11  (-0.13 to -0.08) | <0.001 |  |
|  | Sociodemographic index | | | | | | |  |
|  | High-middle | 991840  (560771 to 1536651) | 339  (148 to 615) | 764136  (443610 to 1171122) | 334  (147 to 599) | -0.11  (-0.14 to -0.08) | <0.001 |  |
|  | High | 800826  (465150 to 1270459) | 399  (180 to 713) | 774103  (456453 to 1209398) | 409  (186 to 723) | 0.06  (0.02 to 0.09) | 0.003 |  |
|  | Low-middle | 955538  (564615 to 1486367) | 269  (118 to 488) | 1516955  (886252 to 2360146) | 273  (119 to 495) | 0.12  (0.08 to 0.17) | <0.001 |  |
|  | Low | 426707  (250692 to 651160) | 282  (122 to 511) | 1052488  (614996 to 1615159) | 290  (126 to 526) | 0.13  (0.1 to 0.15) | <0.001 |  |
|  | Middle | 1764442  (996202 to 2756820) | 316  (135 to 578) | 1782141  (1034369 to 2770873) | 320  (139 to 579) | 0.01  (-0.04 to 0.06) | 0.815 |  |
|  | Region | | | | | | |  |
|  | East Asia | 1063843  (558934 to 1652776) | 268  (112 to 498) | 634788  (369792 to 946476) | 262  (115 to 473) | -0.32 (-0.43 to -0.2) | <0.001 |  |
|  | Oceania | 5445  (3201 to 8215) | 265  (116 to 483) | 10627  (6204 to 16013) | 265  (116 to 482) | 0.04 (0.02 to 0.07) | <0.001 |  |
|  | Central Asia | 78949  (46426 to 122240) | 400  (178 to 711) | 87847  (51464 to 135463) | 399  (178 to 709) | -0.01  (-0.04 to 0.02) | 0.428 |  |
|  | Central Europe | 123760  (72641 to 193757) | 425  (190 to 751) | 78333  (45623 to 123292) | 427  (191 to 755) | 0.06 (0.04 to 0.07) | <0.001 |  |
|  | Eastern Europe | 226490  (133650 to 350561) | 477  (210 to 845) | 155564  (92160 to 238667) | 478  (210 to 847) | -0.06 (-0.09 to -0.03) | <0.001 |  |
|  | High-income Asia Pacific | 175054  (100022 to 279408) | 407  (184 to 741) | 109321  (62444 to 173375) | 408  (184 to 743) | 0.02 (-0.01 to 0.04) | 0.209 |  |
|  | Australasia | 11419  (6599 to 18024) | 429  (102 to 429) | 13582  (7925 to 21101) | 429  (102 to 429) | -0.01 (-0.03 to 0.01) | 0.327 |  |
|  | Western Europe | 323143  (192046 to 510424) | 385  (177 to 693) | 292139  (174688 to 461679) | 402  (184 to 729) | 0.09 (0.03 to 0.15) | 0.003 |  |
|  | Southern Latin America | 48476  (28546 to 74889) | 367  (165 to 660) | 57126  (33261 to 88271) | 367  (165 to 659) | 0.06 (0.05 to 0.06) | <0.001 |  |
|  | High-income North America | 288060  (170970 to 451266) | 459  (208 to 813) | 333658  (198884 to 522924) | 459  (208 to 813) | -0.03 (-0.1 to 0.05) | 0.486 |  |
|  | Caribbean | 47612  (28160 to 73310) | 439  (192 to 787) | 50672  (29910 to 77609) | 438  (192 to 786) | 0.01 (0 to 0.03) | 0.145 |  |
|  | Andean Latin America | 53158  (31744 to 80950) | 439  (192 to 787) | 77009  (45494 to 117395) | 437  (191 to 785) | 0.11 (0.1 to 0.11) | <0.001 |  |
|  | Central Latin America | 262984  (156228 to 406156) | 492  (218 to 887) | 322956  (191457 to 500142) | 488  (217 to 879) | 0.08 (0.07 to 0.08) | <0.001 |  |
|  | Tropical Latin America | 248305  (147247 to 394767) | 525  (231 to 957) | 274367  (160529 to 434992) | 523  (230 to 953) | 0.14 (0.1 to 0.19) | <0.001 |  |
|  | North Africa and Middle East | 409594  (239564 to 640712) | 384  (166 to 696) | 607802  (352698 to 950434) | 375  (162 to 680) | -0.02 (-0.04 to 0) | 0.13 |  |
|  | South Asia | 677582  (403935 to 1052115) | 205  (90 to 374) | 1092159  (645939 to 1698831) | 205  (90 to 375) | 0.06 (-0.01 to 0.13) | 0.11 |  |
|  | Central sub-Saharan Africa | 55898  (32481 to 88677) | 330  (144 to 611) | 144632  (84340 to 229270) | 330  (144 to 611) | -0.01 (-0.02 to -0.01) | <0.001 |  |
|  | Eastern sub-Saharan Africa | 150276  (88548 to 229313) | 250  (108 to 448) | 356391  (208144 to 548156) | 249  (108 to 446) | 0.04 (0.02 to 0.06) | <0.001 |  |
|  | Southern sub-Saharan Africa | 65627  (38549 to 102749) | 389  (170 to 713) | 83386  (48705 to 130284) | 383  (168 to 703) | -0.02 (-0.04 to 0) | 0.11 |  |
|  | Western sub-Saharan Africa | 240379  (141089 to 363954) | 412  (181 to 746) | 646556  (380233 to 981317) | 412  (181 to 744) | -0.01 (-0.02 to 0) | 0.259 |  |
|  | Southeast Asia | 388413  (224244 to 605799) | 264  (115 to 487) | 466039  (265132 to 730996) | 268  (117 to 495) | 0.13 (0.13 to 0.13) | <0.001 |  |
|  | Data in parentheses are 95% uncertainty intervals for cases and incidence, and 95% confidence intervals for AAPCs. | | | | | | |  |
|  |  | | | | | | |  |

Supplementary Table 2: Decomposition analysis of change in YLDs.

|  | **Sex** | **Location** | **Overll difference** | **Aging (percent)** | **Population**  **(percent)** | **Epidemiological change (percent)** |  |
| --- | --- | --- | --- | --- | --- | --- | --- |
|  | **Both** | Global | 336664.7 | -8208.18 (-2.44%) | 364610.03 (108.3%) | -19737.15 (-5.86%) |  |
|  |  | Low SDI | 212807.91 | 3422.63 (1.61%) | 201278 (94.58%) | 8107.28 (3.81%) |  |
|  |  | Low-middle SDI | 195713.45 | 13363.85 (6.83%) | 173507.95 (88.65%) | 8841.64 (4.52%) |  |
|  |  | Middle SDI | 15060.99 | -6392.24 (-42.44%) | 4124.45 (27.38%) | 17328.78 (115.06%) |  |
|  |  | High-middle SDI | -75217.58 | -6716.68 (8.93%) | -67679.98 (89.98%) | -820.92 (1.09%) |  |
|  |  | High SDI | -11711.95 | -1136.69 (9.71%) | -15659.88 (133.71%) | 5084.63 (-43.41%) |  |
|  | **Male** | Global | 139274.69 | -3331.79 (-2.39%) | 166720.87 (119.71%) | -24114.4 (-17.31%) |  |
|  |  | Low SDI | 86201.36 | 1538.95 (1.79%) | 82260.44 (95.43%) | 2401.98 (2.79%) |  |
|  |  | Low-middle SDI | 82855.02 | 5453.54 (6.58%) | 75212.94 (90.78%) | 2188.54 (2.64%) |  |
|  |  | Middle SDI | 8579.9 | -2996.64 (-34.93%) | 6224.51 (72.55%) | 5352.02 (62.38%) |  |
|  |  | High-middle SDI | -32115.69 | -2597.94 (8.09%) | -26705.9 (83.16%) | -2811.84 (8.76%) |  |
|  |  | High SDI | -6244.67 | -298.41 (4.78%) | -6150.91 (98.5%) | 204.65 (-3.28%) |  |
|  | **Female** | Global | 197390.01 | -4877.14 (-2.47%) | 195565.14 (99.08%) | 6702.01 (3.4%) |  |
|  |  | Low SDI | 126606.55 | 1723.27 (1.36%) | 119092.02 (94.06%) | 5791.26 (4.57%) |  |
|  |  | Low-middle SDI | 112858.43 | 7858.83 (6.96%) | 97973.37 (86.81%) | 7026.24 (6.23%) |  |
|  |  | Middle SDI | 6481.1 | -3251.6 (-50.17%) | -3395.45 (-52.39%) | 13128.15 (202.56%) |  |
|  |  | High-middle SDI | -43101.9 | -4240.75 (9.84%) | -42140.6 (97.77%) | 3279.46 (-7.61%) |  |
|  |  | High SDI | -5467.28 | -889.4 (16.27%) | -9771.28 (178.72%) | 5193.4 (-94.99%) |  |
|  | YLDs=years lived with disability. | | | | | |  |
|  |  | | | | | |  |
